# High Throughput Screening of FDA-Approved Drug Library Reveals the Compounds that Promote IRF3-Mediated Pro-Apoptotic Pathway Inhibit Virus Replication

**DOI:** 10.3390/v12040442

**Published:** 2020-04-14

**Authors:** Anna Glanz, Karan Chawla, Stephanie Fabry, Gayatri Subramanian, Julie Garcia, Bryanna Jay, Jacob Ciricillo, Ritu Chakravarti, R. Travis Taylor, Saurabh Chattopadhyay

**Affiliations:** 1 Department of Medical Microbiology and Immunology, University of Toledo College of Medicine, Toledo, OH 43614, USA; Anna.Glanz@rockets.utoledo.edu (A.G.); Karan.Chawla@rockets.utoledo.edu (K.C.); Stephanie.Fabry@rockets.utoledo.edu (S.F.); Gayatri.Subramanian@rockets.utoledo.edu (G.S.); Julie.Gartland@rockets.utoledo.edu (J.G.); Bryanna.Jay@rockets.utoledo.edu (B.J.); Jacob.Ciricillo@rockets.utoledo.edu (J.C.); Travis.Taylor@utoledo.edu (R.T.T.); 2 Department of Physiology and Pharmacology, University of Toledo College of Medicine, Toledo, OH 43614, USA; Ritu.Chakravarti@utoledo.edu

**Keywords:** IRF3, RIPA, antiviral, innate immunity, drug screen, interferon

## Abstract

Interferon (IFN) regulatory factor 3 (IRF3) is the key transcription factor for the induction of IFN and antiviral genes. The absence of antiviral genes in IRF3 deficiency leads to susceptibility to a wide range of viral infections. Previously, we uncovered a function for nontranscriptional IRF3 (nt-IRF3), RLR (RIG-I-like receptor)-induced IRF3-mediated pathway of apoptosis (RIPA), which triggers apoptotic killing of virus-infected cells. Using knock-in mice expressing a transcriptionally inactive, but RIPA-active, IRF3 mutant, we demonstrated the relative contribution of RIPA to host antiviral defense. Given that RIPA is a cellular antiviral pathway, we hypothesized that small molecules that promote RIPA in virus-infected cells would act as antiviral agents. To test this, we conducted a high throughput screen of a library of FDA-approved drugs to identify novel RIPA activators. Our screen identified doxorubicin as a potent RIPA-activating agent. In support of our hypothesis, doxorubicin inhibited the replication of vesicular stomatitis virus, a model rhabdovirus, and its antiviral activity depended on its ability to activate IRF3 in RIPA. Surprisingly, doxorubicin inhibited the transcriptional activity of IRF3. The antiviral activity of doxorubicin was expanded to flavivirus and herpesvirus that also activate IRF3. Mechanistically, doxorubicin promoted RIPA by activating the extracellular signal-regulated kinase (ERK) signaling pathway. Finally, we validated these results using another RIPA-activating compound, pyrvinium pamoate, which showed a similar antiviral effect without affecting the transcriptional activity of IRF3. Therefore, we demonstrate that the RIPA branch of IRF3 can be targeted therapeutically to prevent virus infection.

## 1. Introduction

**Figure 1:**
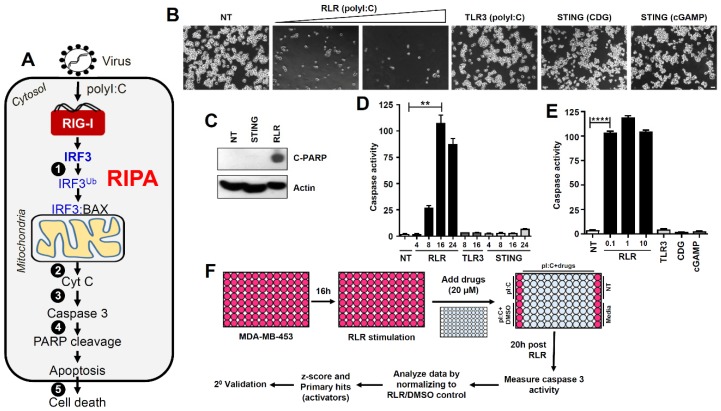
RLR (RIG-I-like receptor)-induced IRF3-mediated pathway of apoptosis (RIPA), but not Toll-like receptor 3 (TLR3) or stimulator of interferon genes (STING)-activated pathways, triggers apoptotic killing in human breast cancer cells. (**A**) Cartoon showing the activation and various stages (indicated by numbers) of RIPA in virus-infected cells. (1) Linear ubiquitination of IRF3 by linear ubiquitin chain assembly complex (LUBAC), (2) release of cytochrome c from mitochondria, (3) activation of executioner caspase 3, (4) cleavage of poly ADP ribose polymerase (PARP), and (5) apoptotic cell death of the virus-infected cell. (**B**) MDA-MB-453 cells were transfected (RLR; 2 and 4 μg) or treated (TLR3; 100 μg/mL) with polyI:C, transfected with STING ligands, cyclic-di-GMP (CDG, 4 μg) or cyclic-GMP-AMP (cGAMP, 4 μg), and the culture fields were photographed after 48 h. Scale bar 100 μm. (**C**) MDA-MB-453 cells were transfected with CDG (STING) or polyI:C (RLR), and C-PARP was measured by immunoblot after 24 h. (**D**) MDA-MB-453 cells were stimulated by TLR3, RLR, or STING ligands (as in B) for the indicated times when caspase-3 activity was measured. (**E**) MDA-MB-453 cells were stimulated by RLR, TLR3 (indicated amounts of polyI:C transfected in μg) or STING ligands for 24 h when caspase-3 activity was measured. (**F**) Our strategy to screen a library of FDA-approved drugs to identify the regulators of RIPA using MDA-MB-453 cells. ** indicates *p* < 0.01, **** *p* < 0.0001.

The innate immune response is the first line of defense against microbial infection. The interferon (IFN) system represents a key antiviral innate immune response mechanism that dictates the outcome of a viral infection [1]. IFN-β, a type-I IFN, is synthesized in the virus-infected cells by the transcriptional activity of IFN regulatory factor 3 (IRF3) [2,3]. IRF3 remains as an inactive monomer in the cytosol of the uninfected cells; upon virus infection, it gets phosphorylated, dimerized, and translocated to the nucleus [2,4]. In the nucleus, the dimeric IRF3 binds to the promoters of IFN-β and many IFN-stimulated genes (ISGs). Secreted IFNs act via autocrine and paracrine signaling to amplify the transcriptional induction of hundreds of ISGs [5]. The ISG-encoded protein products act on specific stages of the virus life cycle to inhibit viral replication and pathogenesis. The absence of antiviral genes in *Irf3*^−/−^ mice causes high susceptibility to a wide range of viruses [6,7]. We have uncovered that, in addition to the transcriptional activity, IRF3 functions in a nontranscriptional (nt) pathway in antiviral defense [8,9,10,11,12,13,14,15]. In contrast to the transcriptional pathway, nt-Irf3 in virus-infected cells functions as a chaperone protein by translocating the pro-apoptotic protein BCL2-associated X (BAX) to the mitochondria, thereby causing apoptotic cell death, which we named RLR (RIG-I-like receptor)-induced IRF3-mediated pathway of apoptosis (RIPA) (Figure 1A) [7,8,9,10,11,12,13]. In RIPA, IRF3 is activated by linear ubiquitination on two key lysine residues by linear ubiquitin chain assembly complex (LUBAC) to allow its interaction with the pro-apoptotic protein BAX [7,12]. The IRF3/BAX complex translocates to the mitochondrial membrane and stimulates the release of cytochrome c into the cytosol. Cytosolic cytochrome c activates cellular caspases to trigger apoptotic cell death (Figure 1A). Recently, we demonstrated that RIPA contributes to the optimal antiviral activity of IRF3. Knock-in mice expressing a RIPA-active nt-Irf3 mutant (*Irf3*^*S1*/*S1*^) can mount antiviral protection against respiratory pathogenesis by Sendai virus (SeV) [7]. The SeV-infected cells, in the absence of RIPA, establish viral persistence [14]. SeV temporarily regulates RIPA by activating the cellular survival pathways, e.g., the phosphatidylinositol 3-kinase (PI3K)-dependent AKT, which stabilizes X-linked inhibitor of apoptosis protein (XIAP), an inhibitor of RIPA [15]. The inhibition of PI3K rapidly triggers RIPA to kill the virus-infected cells [15].

In the time since we described a function for nt-IRF3, several studies have reported RIPA-like activities in viral and nonviral pathogenesis. In human T cell leukemia virus (HTLV1)-infected primary monocytes, stimulator of interferon genes (STING)-activated IRF3 interacts with BAX to cause apoptosis. The IRF3/BAX-mediated monocyte cell death prevents productive HTLV1 replication [16]. In hepatocytes, STING-activated IRF3 causes alcoholic liver diseases (ALD) during chronic ethanol administration in mice [17]. Ethanol administration triggers endoplasmic reticulum stress, which activates STING signaling to enable an interaction between IRF3 and BAX, leading to hepatocyte apoptosis. A subsequent study further revealed that carbon tetrachloride (CCl4)-induced hepatotoxicity is caused by the RIPA-like activity of IRF3, mediated by STING/IRF3/BAX-dependent apoptotic pathway. To further investigate the role of RIPA in ALD, we used the *Irf3*^*S1*/*S1*^ knock-in mice in a mouse alcoholic hepatitis model to show that ethanol administration activates RIPA in hepatic immune cells. Since the immune cells are necessary for the resolution of liver injury, our study demonstrated a detrimental role for RIPA in ALD pathogenesis [18]. In contrast, *Irf3*^*S1*/*S1*^ mice are protected in high-fat diet (HFD)-induced liver diseases by the resolution of hepatic inflammation [19]. The involvement of RIPA in various disease models highlights its potential as a therapeutic target. To test this, we took a pharmacological approach to isolate small molecule modifiers of RIPA. In the current study, we performed a high throughput screen of a library of FDA-approved compounds (Prestwick Chemical), and isolated a small subset of RIPA-promoting compounds. Using two compounds, which specifically activated RIPA, but not the transcriptional function of IRF3, we demonstrated that therapeutic activation of the RIPA branch of IRF3 inhibits virus replication.

## 2. Materials and Methods

### 2.1. Cells, Plasmids, and Reagents

Human cell lines MDA-MB-453 (ATCC HTB-131), HT1080 (ATCC CCL-121), and A549 (ATCC CCL-185), the African green monkey cell line Vero (ATCC CCL-81), and mouse embryonic fibroblasts (MEFs) were maintained in DMEM containing 10% FBS, penicillin, and streptomycin. All cell lines used in this study were maintained in the authors’ laboratory. Expression vectors of human IRF3 and IRF3-K10 were described previously [7], and the ligands for retinoic acid-inducible gene-I (RIG-I), toll-like receptor 3 (TLR3), and STING have been described before [7,20]. The FDA-approved drug library was obtained from Prestwick Chemical (PC, Washington, DC, USA). Individual chemicals were obtained from Sigma-Aldrich (St. Louis, MO, USA) [doxorubicin (Sigma #44583), pyrvinium pamoate (Sigma # P0027)] or from Santa Cruz Biotechnology (Dallas, TX, USA) [U0126 (SC #222395) and SP600125 (SC #200635)]. The antibodies against the specific proteins were obtained as indicated: anti-cleaved PARP (Cell Signaling (Danvers, MA, USA) #9546), anti-phospho-ERK (Cell Signaling #4370), anti-ERK (Cell Signaling #4695), anti-phospho-JNK (Cell Signaling #9251), anti-JNK (Cell Signaling #9252), anti-IRF3 (Santa Cruz #33641), anti-Ub (Santa Cruz #sc-8017), anti-cytochrome c (Santa Cruz #sc-8385), anti-ICP8 (Santa Cruz #53329), anti-β-tubulin (Abcam (Cambridge, MA, USA) #ab15568), anti-ICP0 (Abcam #ab6513), anti-GFP (Roche (Indianapolis, IN, USA) #11814460001), anti-actin (Sigma-Aldrich #A5441), anti-V5 (Thermo Fisher Scientific (Waltham, MA, USA) #R960-25), anti-IFIT1 (described previously [7,9]), anti-IFIT3 (described previously [7,9]), and anti-VSV G-protein (described previously [21,22]).

### 2.2. High-Throughput SCREENING of FDA-Approved Drug Library to Isolate RIPA Activators

The human breast cancer cell line MDA-MB-453 was used to screen the library of FDA-approved drugs to isolate the regulators of RIPA (Figure 1F). The cells were seeded in 96-well black-bottom tissue culture plates, and the next day, the cells were transfected with polyI:C using Lipofectamine 2000 to stimulate RIPA. After the addition of the transfection complex, the cells were immediately treated with either DMSO (vehicle) or the drug library (at 20 μM final concentration). After 20 h of the RIPA stimulation, the cells were analyzed for caspase activity using Apo-ONETM Homogeneous Caspase-3/7 Assay (Promega, San Luis Obispo, CA, USA) following the manufacturer’s instructions. The caspase activity of the vehicle-treated well was arbitrarily considered as 100 and all other values were normalized to this. A representative drug screening plate is shown in Figure S1. The normalized caspase activity was used to calculate the z-scores and the top 25 primary hits were isolated for further validation.

### 2.3. RIPA Stimulation and Drug Treatments

RIPA stimulation was performed by transfecting the cells with polyI:C (1 μg if not indicated otherwise in the figure legends) using Lipofectamine 2000 for 16–24 h, and the cells were either analyzed for caspase activity or cleaved PARP, as indicated in the figure legends. For the drug treatments, the cells were pretreated with the drugs (e.g., doxorubicin at 5 μM, and pyrvinium pamoate at 1 μM or as indicated in the figures) for 1 h before RIPA stimulation (polyI:C transfection). DMSO was used as a vehicle control for these drugs.

### 2.4. Generation of CRISPR/Cas9-Mediated IRF3 Knockout Cells

HT1080 cells were transfected with either control (sc-418922) or IRF3-specific (sc-417171) CRISPR/Cas9 plasmids (Santa Cruz) using Lipofectamine 2000 (Thermo Fisher Scientific). Transfected cells were sorted for high GFP-expressers using flow cytometry, and the GFP-expressing cells were expanded to isolate individual clones. These clones were screened for IRF3 protein levels by immunoblot, and the clones with no IRF3 protein expression were expanded and further validated using functional assay to ensure the absence of IRF3-dependent IFIT3 induction upon RLR stimulation (Figure S2).

### 2.5. Virus Infection and Quantification

Vesicular stomatitis virus (VSV) Indiana strain expressing green fluorescent protein (GFP), Sendai virus (SeV) Cantell strain (Charles River Laboratories, Garfield Heights, OH, USA), herpes simplex virus 1 (HSV-1) KOS and F strains, Langat virus (LGTV), Kunjin virus (KUNV), and the infection procedures have been described previously [13,20,23]. Briefly, the cells were infected with the viruses [at a multiplicity of infection (MOI) of 1] in serum-free DMEM for 2 h, after which the cells were washed and replaced with normal growth medium. The virus-infected cells were analyzed at the indicated time for viral protein expression or as described in figure legends. For quantification of infectious virus particles in the culture medium, plaque assays were performed for VSV in 10-fold serial dilution on Vero cells [24]. HSV-1 titer was measured by TCID50 using 10-fold serial dilution of the culture media on Vero cells [25]. Plaque assays were performed for LGTV and KUNV using previously described procedures [23]. To determine the effects of the drugs on viral replication, the cells were pre-treated with DMEM containing DMSO (vehicle) or individual drugs at the indicated concentrations for 2 h before virus infection, removed during the virus adsorption, and reintroduced post adsorption. Equal protein extracts from the GFP.VSV-infected cells were analyzed for GFP fluorescence using a plate reader. GFP fluorescence of vehicle-treated VSV-infected cells was set arbitrarily at 100 and all other values were normalized to this.

### 2.6. Cell Lysis, Immunoblot, and Ubiquitination Assay

Immunoblot analyses were performed using previously described procedures [7,9]. Briefly, the cells were lysed in 50 mM Tris buffer, pH 7.4, containing 150 mM of NaCl, 0.1% Triton X-100, 1 mM sodium orthovanadate, 10 mM of sodium fluoride, 10 mM of β-glycerophosphate, 5 mM sodium pyrophosphate, protease and phosphatase inhibitors (Roche). Total protein extracts were analyzed by SDS-PAGE followed by immunoblot. For analyzing the ubiquitination of IRF3, a previously described procedure was followed [7]. The immunoblots were quantified by Image J software.

### 2.7. RNA Isolation and qRT-PCR Analyses

Total RNA was isolated using Trizol (Thermo Fisher Scientific), cDNA was prepared using ImProm-II Reverse Transcription Kit (Promega), and the cDNA was analyzed using RadiantTM SYBR Green PCR mix (Alkali Scientific Inc., Fort Lauderdale, FL, USA) in Roche LightCycler 96 instrument and analyzed with the LightCycler 480 Software, Version 1.5. The expression levels of the mRNAs were normalized to 18S rRNA. For the qRT-PCR analyses of the respective genes, the following primers were used:

IFIT1-fwd: TCTCAGAGGAGCCTGGCTAAG,
IFIT1-rev: GTCACCAGACTCCTCACATTTGC,
IFIT3-fwd: GAACATGCTGACCAAGCAGA,
IFIT3-rev: CAGTTGTGTCCACCCTTCCT,
IFNB1-fwd: CGCCGCATTGACCATCTA,
IFNB1-rev: GACATTAGCCAGGAGGTTCT,
18S-fwd: ATTGACGGAAGGGCACCACCAG,
18S-rev: CAAATCGCTCCACCAACTAAGAACG.

### 2.8. Confocal Microscopy

MDA-MB-453 cells were grown on coverslips, infected with HSV-1 in the absence or the presence of doxorubicin, as described in the figure legends. The infected cells were fixed in 4% paraformaldehyde (Electron Microscopy Sciences, Hatfield, PA, USA, #15710), permeabilized in 0.2% Triton X-100 (Fisher Scientific #9002-93-1) and immunostained with anti-ICP0 antibody followed by Alexa.

Fluor-conjugated secondary antibody (Invitrogen #A-11004). The coverslips were mounted on microscopy slides using VectaShield/DAPI (Vector Laboratories, Burlingame, CA, USA, #H-1200) and analyzed using an Olympus (Waltham, MA, USA) confocal microscope and Olympus Fluoview FV1000 software.

### 2.9. Caspase Activity

The caspase-3/7 activity of the cell lysates was performed using previously described procedures [9]. Briefly, the cell lysates were used for measuring caspase activity using the Apo-ONETM Homogeneous Caspase-3/7 Assay according to protocols provided by the manufacturer (Promega). Caspase activity in experimental samples was plotted relative to RLR-stimulated cells arbitrarily set as 100 (as in Figure 1D).

### 2.10. Cell Viability Assays

Cell viability was measured by 3-(4,5-dimethylthiazol-2-yl)-2,5-diphenyltetrazolium bromide (MTT) assay, trypan blue exclusion, and brightfield microscopy. To measure cell viability, the cells were seeded in a 96-well plate, and transfected (RLR) or treated (TLR3) with polyI:C in the absence or the presence of doxorubicin or vehicle (DMSO), as indicated, for 24 h. The MTT assay was performed using previously described procedures [26]. The absorbance of the treated cells was considered as 100, and the other values were normalized to this. For the trypan blue exclusion assay, the treated cells were trypsinized, resuspended in complete DMEM, stained with trypan blue and the live and dead cells were counted using a hemocytometer to calculate percent viability for each treatment.

### 2.11. Statistical Analyses

The statistical analyses were performed using GraphPad Prism 5.02 software. The “*p*” values were calculated using two-tailed, unpaired Student’s t-tests and are shown in the relevant figures. The results presented here are the representatives of at least three biological repeats.

## 3. Results

### 3.1. High Throughput Screen to Identify Small Molecule Activators of the Antiviral RIPA Branch of IRF3

**Figure 2:**
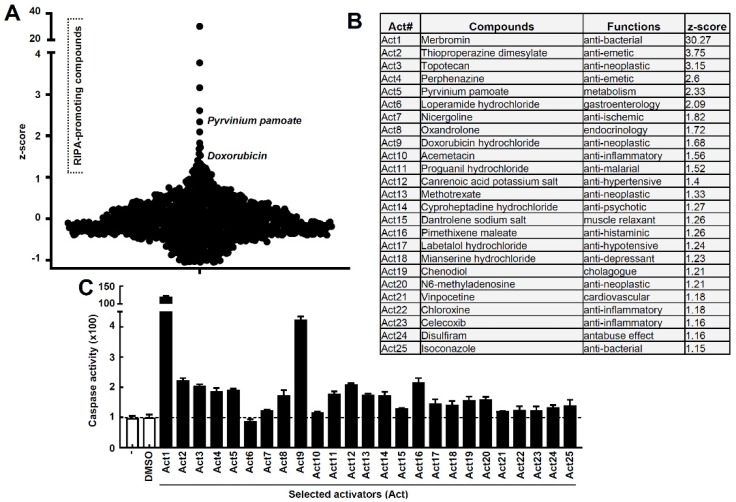
High-throughput screen and validation to isolate RIPA activators. (**A**) Z-scores of the compounds from the primary screen. Caspase activity was normalized to the DMSO control. RIPA-promoting compounds are indicated with z-scores greater than 1. (**B**) Compound names, functions, and corresponding z-scores of the 25 drugs isolated from the primary screen. (**C**) Secondary validation of the primary hits obtained in (**A**). Caspase-3 activity of the selected activators (Act) tested in RLR-stimulated MDA-MB-453 cells normalized to the vehicle (DMSO) control.

To identify small molecule activators of the antiviral RIPA branch of IRF3 (Figure 1A), we performed an unbiased high throughput screen using a library of FDA-approved compounds. Activation of RIPA, by transfection of the RLR ligand polyI:C, caused robust cell death (Figure 1B, stage-5, Figure 1A). Stimulation of TLR3 or STING, by their cognate ligands, did not cause visible cell death (Figure 1B). Cleaved PARP (C-PARP, stage-4, Figure 1A), a molecular marker of apoptosis, was observed in RLR-, but not STING-stimulated, cells. As expected, the apoptotic executioner caspase, caspase-3 (stage-3, Figure 1A), was strongly activated only upon RLR, but not TLR3 or STING, stimulation for an increasing time period (Figure 1D,E). Therefore, we optimized conditions to screen for agents that specifically modulate RIPA-induced apoptosis, without the need to separate the contribution of TLR3 or STING pathways. To isolate the activators of RIPA, we performed a high throughput screen of a library consisting of 1200 FDA-approved compounds [27]. The library is 96-well formatted with each well containing an individual compound. We performed the primary screen using the strategy outlined in Figure 1F, and the activity of caspase-3 was used as a readout of the RIPA activity in each well. The caspase-3 activity of vehicle (DMSO)-treated well was arbitrarily set at 100, and all other values were normalized to this (a sample plate is shown in Figure S1). The primary screen resulted in several RIPA activators, and using an arbitrary cut-off of z-scores greater than 1.15, we obtained twenty-five candidate RIPA activating compounds (Figure 2A,B, Table S1). We noted that the RIPA activators constitute only 2% of the library, indicating the specificities of the compounds. The primary hits were validated using a secondary screen, and the RIPA-promoting activity of each compound was determined with respect to the vehicle control. The secondary screen validated twenty-four of the twenty-five compounds obtained from the primary screen (Figure 2C). The secondary-validated RIPA-activators consisted of compounds from a variety of therapeutic activities, e.g., anticancer, antibacterial, anti-inflammatory, antihypertensive, etc (Figure 2B). We excluded Act1, which triggered caspase-3 activity nonspecifically, from the subsequent studies, and focused on doxorubicin, which has earlier been studied to activate IRF3 [28].

### 3.2. The RIPA-Promoting Agent Doxorubicin Inhibits Vesicular Stomatitis Virus (VSV) Replication in an IRF3-Dependent Manner

**Figure 3:**
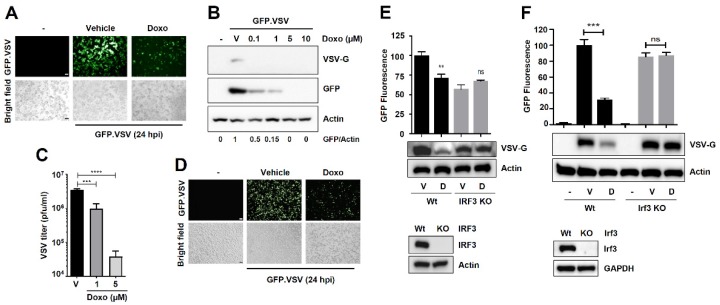
Inhibition of vesicular stomatitis virus (VSV) replication by the RIPA-promoting compound doxorubicin is dependent on IRF3. (**A**) MDA-MB-453 cells were infected with GFP.VSV in the absence or presence of doxorubicin. Culture fields were photographed 24 h post-infection. Scale bar 100 μm. (**B**) MDA-MB-453 cells were infected with GFP.VSV in the absence or presence of doxorubicin (Doxo, at the indicated doses). The expression of VSV G-protein and virus-encoded GFP were analyzed by immunoblot. (**C**) MDA-MB-453 cells were infected with GFP.VSV in the absence or presence of doxorubicin (at the indicated doses). Infectious virion release was analyzed by plaque assay. (**D**) A549 cells were infected with GFP.VSV in the absence or presence of doxorubicin. Culture fields were photographed 24 h post-infection. Scale bar 100 μm. (**E**) Wt or *IRF3*^−/−^ (KO) HT1080 cells were infected with GFP.VSV—in the absence or the presence of doxorubicin (1 μM), and the viral protein (VSV-G) expression was measured by immunoblot, and the VSV-encoded GFP fluorescence was quantified, 24 hpi. Lower panel indicates an immunoblot for IRF3 expression. (**F**) Wt or *Irf3*^−/−^ (KO) MEFs were infected with GFP.VSV in the absence or the presence of doxorubicin (1 μM), and the viral protein (VSV-G) expression was measured by immunoblot, and the VSV-encoded GFP fluorescence was quantified, 24 hpi. D, Doxo, V, Vehicle (DMSO treatment), ** indicates *p* < 0.01, *** *p* < 0.001, **** *p* < 0.0001, ns, not significant.

Because RIPA contributes to the antiviral function of IRF3 [7], we hypothesized that agents that promote RIPA would inhibit viral replication. For this purpose, we used vesicular stomatitis virus (VSV) as a model negative-sense RNA virus. Doxorubicin treatment inhibited VSV replication, analyzed by virus-encoded GFP expression (Figure 3A). We validated these results by immunoblot analyses, which demonstrate that doxorubicin treatment inhibited the expression of VSV-G protein, a viral envelope glycoprotein as well as the virus-encoded GFP (Figure 3B). As expected, the reduced viral protein expression led to reduced infectious virus particle release from doxorubicin-treated cells (Figure 3C). We validated the results in another cell line (A549), in which doxorubicin also inhibited VSV-encoded GFP expression (Figure 3D). To determine whether the antiviral activity of doxorubicin is dependent on IRF3, we generated *IRF3*^−/−^ human cells (HT1080) using a CRISPR/Cas9 approach (Figure 3E, lower panel, Figure S2). Doxorubicin, as expected, inhibited the viral protein expression in the Wt cells; however, the antiviral activity of doxorubicin was impaired in *IRF3*^−/−^ cells (Figure 3E, top panel). To further validate this striking result, we used Wt and *Irf3*^−/−^ mouse embryonic fibroblasts (MEFs) (Figure 3F, lower panel). Similar to the human cells, doxorubicin inhibited VSV replication only in the presence of Irf3 (Figure 3F, top panel). We further quantified, in addition to viral G protein, the expression of VSV-encoded GFP fluorescence. Similar to the inhibition of G protein expression, doxorubicin inhibited GFP fluorescence in Wt but not IRF3-deficient cells (Figure 3E,F). We ensured that doxorubicin did not cause substantial cytotoxicity under our experimental conditions (Figure S3A,B). Our results demonstrate, for the first time, that doxorubicin is a RIPA-promoting agent, which inhibits viral infection in an IRF3-dependent manner.

### 3.3. Doxorubicin Inhibits the Transcriptional Activity of IRF3

**Figure 4:**
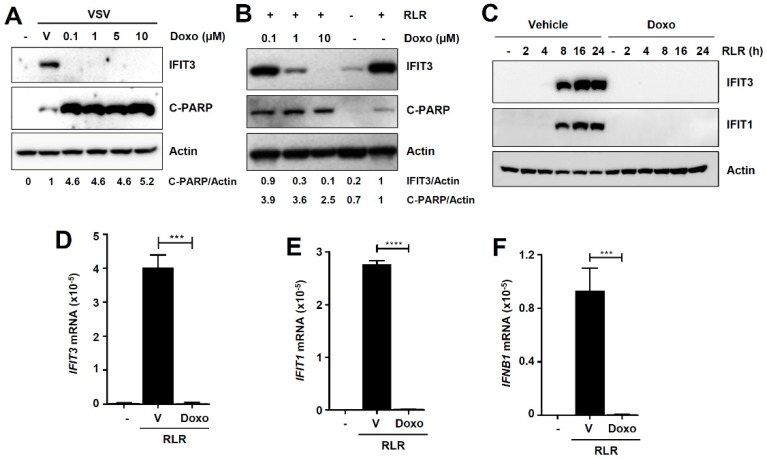
Doxorubicin inhibits the transcriptional activity of IRF3. (**A**) MDA-MB-453 cells were infected with GFP.VSV in the absence or presence of doxorubicin (Doxo, at the indicated doses). IFIT3 and C-PARP were analyzed by immunoblot. (**B**) HT1080 cells were transfected with polyI:C (RLR) in the absence or presence of doxorubicin (Doxo, at the indicated doses). IFIT3 and C-PARP were analyzed by immunoblot 16 h post-RLR stimulation. (**C**) MDA-MB-453 cells were transfected with polyI:C (RLR) for the indicated times in the absence or presence of doxorubicin, IFIT1 and IFIT3 proteins were analyzed by immunoblot. (**D**–**F**) MDA-MB-453 cells were transfected with polyI:C (RLR) in the absence or the presence of doxorubicin. *IFIT3* (**D**), *IFIT1* (**E**), and *IFNB1* (**F**) mRNA levels were analyzed by qRT-PCR analyses 8 h post-RLR. Vehicle (V), DMSO treatment, *** indicates *p* < 0.001, **** *p* < 0.0001.

The optimal antiviral action of IRF3 depends on its transcriptional and nontranscriptional (nt, RIPA) functions. We sought to determine whether doxorubicin activates either one or both functions of IRF3. Furthermore, doxorubicin has previously been reported to induce phosphorylation and transcriptional activation of IRF3 [28]. Thus, we examined whether doxorubicin promotes the transcriptional activity of IRF3 by RLR-signaling in VSV-infected cells. VSV infection induced IRF3-target gene, IFIT3, which was strongly inhibited by doxorubicin, examined at multiple doses (Figure 4A). In contrast, doxorubicin robustly enhanced the RIPA activity, i.e., the C-PARP levels in VSV-infected cells (Figure 4A). To solidify these results, we used a nonviral RLR agonist, polyI:C, and the IRF3-induced IFIT3 was also strongly inhibited by doxorubicin in HT1080 cells (Figure 4B). Similar to VSV-infected cells, doxorubicin promoted IRF3-induced RIPA activity in these cells (C-PARP, Figure 4B). To validate these results, we measured the transcriptional induction of additional IRF3-induced genes, both at the protein and mRNA levels. The IRF3-induced IFIT3 and IFIT1 proteins were strongly inhibited by doxorubicin at multiple times post RLR-stimulation (Figure 4C). Doxorubicin treatment also significantly inhibited the mRNA induction of *IFIT3* (Figure 4D), *IFIT1* (Figure 4E), and *IFN-β* (Figure 4F), all of which depend on the transcriptional activity of IRF3. Collectively, our results demonstrate that doxorubicin inhibits VSV replication in the absence of IRF3-induced antiviral gene expression.

### 3.4. Doxorubicin Is a Newly Identified Activator of the RIPA Branch of IRF3

**Figure 5:**
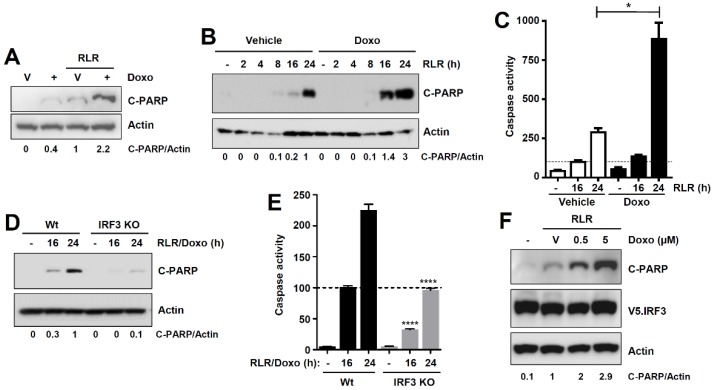
Doxorubicin is a novel activator of the RIPA branch of IRF3. (**A**) MDA-MB-453 cells were transfected with polyI:C in the absence or the presence of doxorubicin for 16 hrs, when C-PARP was analyzed by immunoblot. (**B**) MDA-MB-453 cells were transfected with polyI:C (RLR) in the absence or the presence of doxorubicin for the indicated times, when C-PARP was measured by immunoblot. (**C**) MDA-MB-453 cells were transfected with polyI:C (RLR, as in A) in the absence or presence of doxorubicin for the indicated times, when caspase-3 activity was measured. (**D**) Wt and *IRF3*^−/−^ (KO) HT1080 cells were transfected with polyI:C (RLR) in the presence of doxorubicin for the indicated times, when C-PARP was analyzed by immunoblot. (**E**) Wt and *IRF3*^−/−^ (KO) HT1080 cells were transfected with polyI:C (RLR) in the presence of doxorubicin, and caspase-3 activity was measured at the indicated times post-RLR stimulation. (**F**) *IRF3*^−/−^ cells complemented with IRF3 K10 mutant were transfected with polyI:C (RLR) in the absence or the presence of doxorubicin (at the indicated doses) and C-PARP was measured by immunoblot 24 h post-RLR. Vehicle, DMSO treatment, * indicates *p* < 0.05, **** *p* < 0.0001.

Doxorubicin promoted the RIPA activity, measured by robust increase in RLR-induced C-PARP levels, when compared with RLR-treated cells (Figure 5A,B). Doxorubicin is a known inducer of the cellular apoptotic pathway [29,30], and, as expected, also triggered apoptosis in the absence of RLR stimulation (Figure 5A, lane 2). To inquire genetically how doxorubicin activates IRF3 to promote RIPA, we took a strategy to measure the “doxorubicin-promoted RIPA activity” using the combined treatment of RLR and doxorubicin (“RLR/doxo”, lane 4, Figure 5A), to avoid the individual cellular effects of RLR or doxorubicin (lanes 2, 3, Figure 5A). Similar to C-PARP, doxorubicin significantly increased the RLR-induced caspase-3 activity (Figure 5C). The increased RIPA activity by doxorubicin, as expected, led to enhanced cell death in a dose-dependent manner (Figure S3C). We examined doxorubicin-promoted RIPA in Wt and *IRF3*^−/−^ cells. Doxorubicin caused robust increase in RLR-induced C-PARP and capsase-3 activation in Wt cells (Figure 5D,E). The RLR/doxorubicin-induced C-PARP and caspase activity were reduced in *IRF3*^−/−^ cells (Figure 5D,E). Because doxorubicin specifically promotes the RIPA, and not the transcriptional activity of IRF3, we used a nt-IRF3 mutant, IRF3-K10, which is functional only in the RIPA, but not the transcriptional branch [7]. The IRF3-K10-expressing cells showed strong promotion of RIPA activity upon doxorubicin treatment (Figure 5F). Collectively, our results demonstrate that doxorubicin inhibits the transcriptional, but promotes the RIPA, activity of IRF3.

### 3.5. Doxorubicin Promotes RIPA by Activating the ERK Signaling Pathway

**Figure 6:**
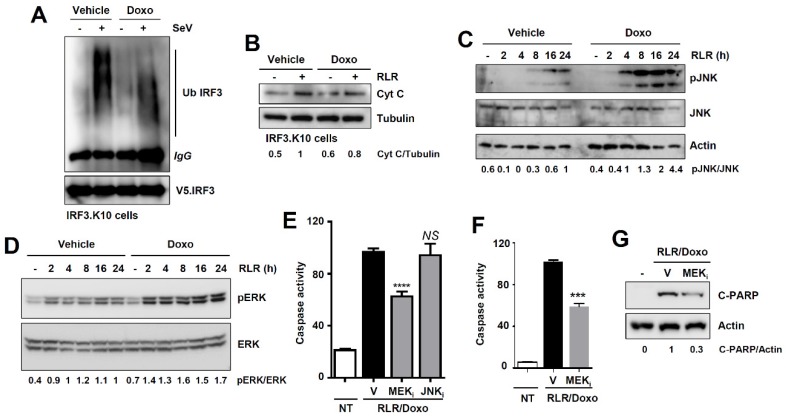
Doxorubicin promotes RIPA by activating the extracellular signal-regulated kinase (ERK) signaling pathway. (**A**) *IRF3^−/−^* cells expressing IRF3 K10 mutant were infected with Sendai virus (SeV) in the absence or the presence of doxorubicin, and ubiquitination of IRF3 was analyzed. (**B**) *IRF3*^−/−^ cells expressing IRF3 K10 mutant were transfected with polyI:C (RLR) in the absence or presence of doxorubicin. The mitochondria-free cytosol was analyzed for cytochrome c (Cyt C) by immunoblot. (**C**,**D**) MDA-MB-453 cells were transfected with polyI:C (RLR) in the absence or presence of doxorubicin for the indicated times, when phosphorylated c-Jun N-terminal kinase (pJNK) (**C**) or pERK (**D**) were analyzed by immunoblot. (**E**) MDA-MB-453 cells were transfected with polyI:C (RLR) in the presence of doxorubicin and treated with either vehicle (V) or the inhibitors of mitogen-activated protein kinase kinase (MEK) (MEK_i_) or JNK (JNK_i_), and caspase-3 activity was measured. (**F**,**G**) *Irf3*^*S1*/*S1*^ MEFs were transfected with polyI:C (RLR) in the presence of doxorubicin, and treated with either vehicle (V) or the inhibitor of MEK (MEK_i_); caspase-3 activity (**F**) and C-PARP (**G**) were analyzed 16 h post-RLR. Vehicle (V), DMSO treatment, *** indicates *p* < 0.001, **** *p* < 0.0001, NS, not significant.

To investigate the biochemical mechanism by which doxorubicin promotes RIPA, we examined whether doxorubicin activates any specific step(s) of RIPA (depicted in Figure 1A). We systematically analyzed both pre- and postmitochondrial steps of RIPA. The ubiquitination of IRF3 (stage-1, Figure 1A), which is essential for activating RIPA, was not promoted by doxorubicin (Figure 6A). We noted that doxorubicin-treated cells exhibited shorter ubiquitin chains linked to IRF3 than the vehicle-treated cells. The results led us to examine whether doxorubicin increased the RLR-induced release of cytochrome c into the cytosol (stage-2, Figure 1A), a step that requires the interaction of IRF3 and BAX. Doxorubicin did not increase the RLR-induced release of cytosolic cytochrome c (Figure 6B). These results led us to hypothesize that doxorubicin activates RLR-activated cellular signaling to promote RIPA. A previous study indicated that p53, which can be activated by doxorubicin [24], is not involved in RLR-induced apoptosis [31]. Therefore, we examined the c-Jun N-terminal kinase (JNK) and extracellular signal-regulated kinase (ERK) signaling pathways, which are activated upon RLR stimulation [32,33]. RLR stimulation triggered the phosphorylation of JNK (pJNK) and ERK (pERK), which were enhanced by doxorubicin (Figure 6C,D). We then tested whether doxorubicin-activated JNK and ERK are involved in promoting RIPA, using pharmacological inhibitors of JNK and mitogen-activated protein kinase kinase (MEK), the upstream activator of ERK. Inhibition of MEK (U0126, MEK_i_), but not JNK (SP600125, JNK_i_), significantly suppressed doxorubicin-promoted RIPA (Figure 6E). We further validated the role of ERK in mouse cells expressing the nt-Irf3 mutant (*Irf3*^*S1*/*S1*^), which is active in RIPA [7]. In *Irf3*^*S1*/*S1*^ MEFs, the MEK inhibitor suppressed doxorubicin-promoted RIPA, analyzed by RLR-induced caspase-3 activity (Figure 6F) and C-PARP (Figure 6G). Together, doxorubicin-activated ERK signaling is involved in promoting RIPA.

### 3.6. Doxorubicin Inhibits Flavivirus and Herpesvirus Replication

**Figure 7:**
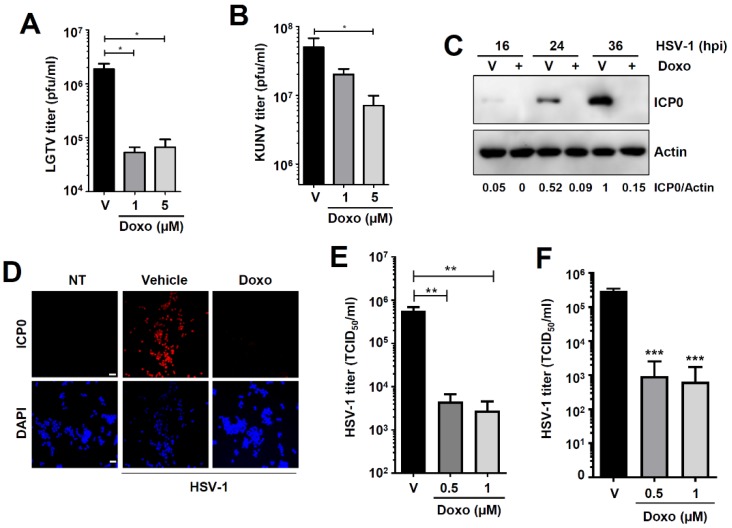
Doxorubicin inhibits flavivirus and herpes simplex virus 1 (HSV-1) replication. (**A**,**B**) Vero cells were infected with Langat virus (LGTV) (**A**) or Kunjin virus (KUNV) (**B**) in the absence or the presence of doxorubicin (Doxo, at the indicated doses). Virion production was analyzed by plaque assay. (**C**) MDA-MB-453 cells were infected with HSV-1 in the absence or the presence of doxorubicin. The expression of viral protein infected cell protein 0 (ICP0) was analyzed by immunoblot at the indicated times post-infection. (**D**) MDA-MB-453 cells were infected with HSV-1 in the absence or the presence of doxorubicin. The expression of viral ICP0 protein was analyzed by confocal microscopy 24 h post-infection. Scale bar 50 μm. (**E**,**F**) MDA-MB-453 cells were infected with HSV-1 (KOS strain, E or F strain, F) in the absence or the presence of doxorubicin for 24 h when the infectious virion release was analyzed by TCID50 assay. Vehicle (V), DMSO treatment, * indicates *p* < 0.05, ** *p* < 0.01, *** *p* < 0.001.

To test whether doxorubicin inhibits viruses of other families, we used members of *Flaviviridae* and *Herpesviridae*, which are known to activate the RLR signaling pathway [34,35,36]. We previously demonstrated that RIPA can be activated by viruses with positive-sense RNA or DNA genomes [13]. Consistent with the VSV results (Figure 3), doxorubicin treatment inhibited Langat virus (LGTV) (Figure 7A) and Kunjin virus (KUNV) (Figure 7B) infectious virion production. Furthermore, cytosolic DNA from DNA viruses can activate RIPA through RNA polymerase III activity [13]. To investigate whether doxorubicin also inhibits DNA virus replication, we used two strains (KOS and F) of HSV-1. Doxorubicin strongly inhibited HSV-1 (KOS strain) replication, indicated by the reduction in ICP0 expression by immunoblot (Figure 7C) and confocal microscopy (Figure 7D). The inhibition of viral protein expression led to a significant reduction in infectious virus particle release from the doxorubicin-treated cells (Figure 7E). Similar to the KOS strain, doxorubicin also inhibited the replication of HSV-1 F strain (Figure 7F). Collectively, our results demonstrate that the RIPA-promoting agent doxorubicin inhibits the replication of both RNA and DNA viruses.

### 3.7. Pyrvinium Pamoate, Another RIPA-Promoting Compound Inhibits Virus Replication without Affecting the Transcriptional Activity of IRF3

**Figure 8:**
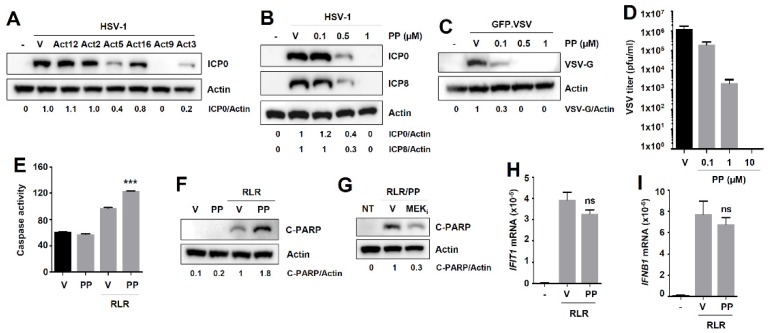
Another RIPA-promoting drug, pyrvinium pamoate, is antiviral against VSV and HSV-1, without affecting IFN and ISG production. (**A**) HT1080 cells were infected with HSV-1 in the absence or the presence of the selected RIPA-promoters (Act12, Act2, Act5, Act16, Act9, and Act3). Viral protein ICP0 was analyzed by immunoblot 24 h post-infection. (**B**) HT1080 cells were infected with HSV-1 in the absence or the presence of pyrvinium pamoate (PP, at the indicated doses) and expression of viral proteins (ICP0 and ICP8) was analyzed by immunoblot 24 h post-infection. (**C**) HT1080 cells were infected with GFP.VSV in the absence or presence of pyrvinium pamoate (PP, at the indicated doses), and VSV-G protein expression was analyzed by immunoblot 24 h post-infection. (**D**) HT1080 cells were infected with GFP.VSV in the absence or presence of pyrvinium pamoate (PP, at the indicated doses), and virus titer was determined via plaque assay in Vero cells 24 h post-infection. (**E**,**F**) HT1080 cells were transfected with polyI:C (RLR) in the absence or the presence of pyrvinium pamoate, and caspase-3 activity (**E**) and C-PARP (**F**) were measured 16 h post-RLR. (**G**) HT1080 cells were transfected with polyI:C (RLR) in the presence of pyrvinium pamoate, and treated with either vehicle (V) or the inhibitor of MEK (MEK_i_), and C-PARP was analyzed 16 h post-RLR. (**H**,**I**) HT1080 cells were transfected with polyI:C (RLR) in the absence or the presence of pyrvinium pamoate, and the mRNA expression of IRF3-dependent genes *IFIT1* (**H**) and *IFNB1* (**I**) were analyzed by qRT-PCR 8 h post-RLR. Vehicle (V), DMSO treatment, *** indicates *p* < 0.001, ns, not significant (*p* > 0.05).

To test the generality of the theme that RIPA-promoting compounds are antiviral agents, we screened a subset of the secondary-validated RIPA-promoters (Act12, Act2, Act5, Act16, Act9, and Act3), based on an arbitrary cut-off of a 1.9-fold increase in RIPA-promotion (Figure 2C), for their ability to inhibit HSV-1 replication (Figure 8A). We confirmed the anti-HSV-1 activity of doxorubicin (Act9), and also isolated two additional compounds (Act3 and Act5) that strongly inhibited ICP0 expression (Figure 8A). We excluded Act3 (topotecan) from further analyses because it shares properties with doxorubicin and may activate similar pathways. We focused on the anti-helminthic drug pyrvinium pamoate (PP, Act5) for validation of our hypothesis. Similar to doxorubicin, PP inhibited the replication of HSV-1 in HT1080 cells, in a dose-dependent manner, demonstrated by immunoblot of viral proteins ICP0 and ICP8 (Figure 8B). PP also inhibited VSV replication, analyzed by immunoblot of the viral protein VSV-G (Figure 8C) and infectious virion particle release (Figure 8D). The antiviral activity of PP prompted us to test its ability to activate IRF3. PP, as expected, promoted the RIPA branch of IRF3, measured by caspase activity (Figure 8E) and C-PARP (Figure 8F). Unlike doxorubicin, PP treatment did not trigger an apoptotic pathway in the absence of RLR-stimulation (Figure 8F, lane 2). To investigate whether PP uses a mechanism similar to doxorubicin to promote RIPA, we tested the MEK inhibitor in PP-promoted RIPA. Inhibition of MEK reduced RLR-induced C-PARP in PP-treated cells (Figure 8G). Therefore, ERK signaling may be a common mechanism to therapeutically target RIPA in multiple cell types. Next, we inquired about the effect of PP on the transcriptional activity of IRF3. Interestingly, although PP promoted the RIPA branch of IRF3 (Figure 8E,F), it had no effect on the transcriptional activity of IRF3, demonstrated by qRT-PCR analyses of IRF3 target genes (Figure 8H,I). Therefore, in contrast to doxorubicin, which differentially modulates IRF3 activity by promoting RIPA and inhibiting the IRF3 transcriptional branch, PP exclusively promotes RIPA without affecting IRF3 transcriptional activity.

## 4. Discussion

We have previously demonstrated, using genetically manipulated cells and mice, that nt-IRF3 function via RIPA contributes to the optimal antiviral activity of IRF3 [7,8,9,10,12]. In the current study, we sought to identify small molecules that activate RIPA and, therefore, can limit virus replication. Using a high-throughput screen of a library of FDA-approved drugs, we isolated a subset of compounds with diverse therapeutic functions that promoted the activity of RIPA. Among the RIPA-activating compounds, we focused on doxorubicin for mechanistic and functional studies. Doxorubicin inhibits the replication of VSV by activating the RIPA branch of IRF3. Our in-depth biochemical studies revealed that doxorubicin inhibited the transcriptional activity of IRF3; moreover, doxorubicin promoted RIPA by activating the ERK signaling pathway. The antiviral function of doxorubicin was expanded to the tick- and mosquito-borne flaviviruses (LGTV and KUNV), and HSV-1. Finally, we validated our results using pyrvinium pamoate, which also inhibited the replication of VSV and HSV-1 by specifically promoting RIPA, and not the transcriptional, function of IRF3. Therefore, we demonstrate that compounds that activate RIPA have potential for antiviral action.

High throughput screening to isolate novel antiviral compounds is a widely accepted strategy, and commonly utilizes virus replication as a terminal readout. We took a novel approach to isolate the antiviral agents by screening for activators of a cellular antiviral pathway. IRF3 is the key transcription factor for the induction of IFN and antiviral ISGs [1,4,37,38]. We have uncovered a pro-apoptotic pathway of nt-IRF3, RIPA, in virus-infected cells [7,9,13]. Using cells and mice expressing a RIPA active nt-IRF3 mutant, we demonstrated the relative contribution of RIPA to the antiviral response of IRF3 [7]. Several high throughput screens have revealed small molecules that regulate the transcriptional activity of IRF3 [39,40,41,42,43]. However, because RIPA is newly identified, there are no known regulators of this pathway. This led us to develop an unbiased screen, using a library of FDA-approved drugs, to isolate RIPA-activators that would function as antiviral agents. Using doxorubicin, a new RIPA activator, we examined both activities of IRF3, because an activator of either pathway would provide an antiviral response. Although doxorubicin promoted RIPA in multiple cell types, it inhibited the transcriptional activity of IRF3. Therefore, doxorubicin inhibits virus replication in the absence of IRF3-induced antiviral genes, further emphasizing the biological significance of RIPA. How doxorubicin inhibits the transcriptional activity of IRF3 is a topic of future investigation. The DNA-damaging property of doxorubicin [44] may contribute to this activity. Furthermore, whether doxorubicin, in addition to RIPA promotion, can intercalate with viral genomic DNA to inhibit viral replication, will require further studies. Our screen isolated, in addition to doxorubicin, pyrvinium pamoate, which specifically promoted RIPA, but not the transcriptional function, of IRF3. Many viruses block the transcriptional activity of IRF3 to dampen IFN production [45]. In such scenarios, it will be interesting to examine whether RIPA-promoting compounds inhibit virus replication.

To investigate the molecular mechanisms of the doxorubicin-mediated promotion of RIPA, we examined the key biochemical steps of the pathway. Since viruses can block host pathways, we chose to use nonviral stimuli of RLR for the mechanistic studies, to avoid any virus-specific effects. Our results indicate that doxorubicin did not enhance the IRF3 ubiquitination nor the release of cytochrome c into the cytosol, the critical stages of RIPA. Interestingly, our results suggest that doxorubicin treatment may enrich a distinct pool of ubiquitinated IRF3 in the RLR-stimulated cells. Targeted proteomic analyses of this pool of IRF3 will be done in the future to gain further insight. We also considered that doxorubicin, a DNA-damaging agent, might stimulate RIPA by triggering the STING signaling pathway via topoisomerase activation [46,47]. The cGAS/STING pathway can also trigger transcription-independent mitotic cell death by IRF3 activation [48]. Moreover, ER stress-activated STING has been shown to trigger a RIPA-like pathway in hepatocytes [17]. However, in our test cells, MDA-MB-453, the STING agonists were unable to trigger apoptotic cell death, indicating a lack of STING contribution in promoting RIPA. It remains to be seen whether the RIPA activators also trigger additional innate signaling pathways, e.g., NLRs to amplify RIPA activity. Our results revealed a novel role of the ERK signaling pathway in regulating RIPA. SeV infection activates cellular autophagy via the ERK signaling pathway to trigger apoptosis [49]. Whether doxorubicin or pyrvinium pamoate use a similar mechanism to promote RIPA will require additional studies. Our results further revealed that doxorubicin activated both ERK and JNK signaling pathways in RLR-stimulated cells; however, JNK activity was not required for promoting RIPA. In the future, in-depth genetic studies will reveal the relative contribution of ERK and JNK to modulate RIPA. The ERK signaling pathway also regulates the transcriptional activity of IRF3 [50]; whether doxorubicin inhibits the transcriptional activity of IRF3 via ERK will require future investigation. In the future, in vivo studies in the context of virus-infection will be required to validate the in vitro findings regarding the ERK signaling mechanism of RIPA-activating drugs. A previous study indicated that p53, which can be activated by doxorubicin, is not involved in dsRNA-induced cell death [31]. In previous studies, we have shown that SeV-induced RIPA is regulated by the PI3K/AKT signaling pathway [15]. It remains to be seen, using nt-IRF3 mutant that specifically activates RIPA, whether doxorubicin also modulates the p53 and PI3K pathways to promote RIPA.

Our screen, using a relatively small library of 1200 compounds, revealed that a number of molecules with diverse therapeutic activities promote RIPA. In the future, we will expand the screen to larger libraries to isolate additional novel RIPA-activating compounds. In addition, a screen using cells expressing nt-IRF3 mutant that specifically activates RIPA may reveal additional candidates. In the future, we will use mice expressing a RIPA-active *Irf3*^*S1*/*S1*^ mutant to test the efficacy of the RIPA-promoting drugs in mounting antiviral responses in the absence of antiviral genes. Such studies will be physiologically significant by using the drug derivatives that are currently applied on patients. Because doxorubicin inhibits the cellular IFN response, patients receiving this drug may be protected from the adverse effects of IFN and the IFN-induced genes. Moreover, our study provides a molecular basis for the previously identified antiviral effects of the RIPA-activating compounds [51,52]. A recent study showed that PP is a novel inhibitor of coronavirus replication [53] and its antiviral property, in part, may be related to the RIPA-promoting function of PP. Whether activation of RIPA directly inhibits virus replication or eliminates the virus-infected cells to reduce overall infection requires in-depth studies. Some viruses trigger apoptosis for successful spreading and, therefore, promoting viral apoptosis to inhibit virus infection may be virus specific.

Finally, a strategy to repurpose RIPA activators as antivirals must be met with caution, as there are a number of diseases where RIPA can be detrimental (e.g., liver diseases). For instance, a RIPA-like pathway in hepatocytes leads to the development of alcoholic liver diseases [17]. Moreover, we recently showed that RIPA-activated hepatic myeloid cells produce inflammatory cytokines that cause hepatitis in mice [18]. In these scenarios, it may be advantageous to investigate RIPA inhibitors as therapeutic options. In summary, our results demonstrate that pharmacological activation of RIPA may be a potential antiviral strategy, particularly in scenarios where the cellular IFN response is dampened. In the future, these RIPA-modulating agents can be studied in additional models where RIPA is involved, e.g., in fatty liver diseases.

## Supplementary Materials

The following are available online at https://www.mdpi.com/1999-4915/12/4/442/s1. Figure S1. Representative data from the primary screen. A representative primary screening plate showing the caspase-3 activity in each well (rows and columns are shown in blue) containing a specific drug or DMSO (vehicle) control. Potential activator and inhibitor drugs are shown in the sample plate. NT, no treatment. Figure S2. Validation of IRF3−/− HT1080 cells. Immunoblot of IFIT3 protein expression in RLR-stimulated Wt and IRF3−/− (KO) HT1080 cells to ensure the gene knockout. Figure S3. Cell viability of doxorubicin-treated cells in the absence or the presence of RLR stimulation. (A, B) HT1080 cells were treated with doxorubicin at the indicated concentrations. Cell viability was assessed 16 h post-treatment by trypan blue exclusion assay (A) or brightfield microscopy (B, Scale bar 100 µm). (C) MDA-MB-453 cells were treated with doxorubicin at the indicated concentrations and transfected (RLR) or treated (TLR3) with polyI:C. Cell viability was assessed 24 h post-treatment by MTT assay. NT, no treatment, V, vehicle (DMSO). Table S1. Caspase activity of the primary screening plates. MDA-MB-453 cells were transfected with polyI:C in the absence or the presence of the drugs, as described in Figure 1F. Caspase-3 activity of each well is indicated after normalizing to the vehicle (DMSO) control, which was considered as 100.

## References

[1] Fensterl V., Chattopadhyay S., Sen G.C. (2015). No Love Lost Between Viruses and Interferons. Annu. Rev. Virol..

[2] Ikushima H., Negishi H., Taniguchi T. (2013). The IRF family transcription factors at the interface of innate and adaptive immune responses. Cold Spring Harb. Symp. Quant. Biol..

[3] Jefferies C.A. (2019). Regulating IRFs in IFN Driven Disease. Front. Immunol..

[4] Hiscott J. (2007). Convergence of the NF-kappaB and IRF pathways in the regulation of the innate antiviral response. Cytokine Growth Factor Rev..

[5] Schoggins J.W., Rice C.M. (2011). Interferon-stimulated genes and their antiviral effector functions. Curr. Opin. Virol..

[6] Chattopadhyay S., Fensterl V., Zhang Y., Veleeparambil M., Wetzel J.L., Sen G.C. (2013). Inhibition of viral pathogenesis and promotion of the septic shock response to bacterial infection by IRF-3 are regulated by the acetylation and phosphorylation of its coactivators. MBio.

[7] Chattopadhyay S., Kuzmanovic T., Zhang Y., Wetzel J.L., Sen G.C. (2016). Ubiquitination of the Transcription Factor IRF-3 Activates RIPA, the Apoptotic Pathway that Protects Mice from Viral Pathogenesis. Immunity.

[8] Chattopadhyay S., Fensterl V., Zhang Y., Veleeparambil M., Yamashita M., Sen G.C. (2013). Role of interferon regulatory factor 3-mediated apoptosis in the establishment and maintenance of persistent infection by Sendai virus. J. Virol..

[9] Chattopadhyay S., Marques J.T., Yamashita M., Peters K.L., Smith K., Desai A., Williams B.R., Sen G.C. (2010). Viral apoptosis is induced by IRF-3-mediated activation of Bax. EMBO J..

[10] Chattopadhyay S., Sen G.C. (2010). IRF-3 and Bax: A deadly affair. Cell Cycle.

[11] Chattopadhyay S., Sen G.C. (2014). dsRNA-activation of TLR3 and RLR signaling: Gene induction-dependent and independent effects. J. Interferon Cytokine Res..

[12] Chattopadhyay S., Sen G.C. (2017). RIG-I-like receptor-induced IRF3 mediated pathway of apoptosis (RIPA): A new antiviral pathway. Protein Cell.

[13] Chattopadhyay S., Yamashita M., Zhang Y., Sen G.C. (2011). The IRF-3/Bax-mediated apoptotic pathway, activated by viral cytoplasmic RNA and DNA, inhibits virus replication. J. Virol..

[14] Peters K., Chattopadhyay S., Sen G.C. (2008). IRF-3 activation by Sendai virus infection is required for cellular apoptosis and avoidance of persistence. J. Virol..

[15] White C.L., Chattopadhyay S., Sen G.C. (2011). Phosphatidylinositol 3-kinase signaling delays sendai virus-induced apoptosis by preventing XIAP degradation. J. Virol..

[16] Sze A., Belgnaoui S.M., Olagnier D., Lin R., Hiscott J., van Grevenynghe J. (2013). Host restriction factor SAMHD1 limits human T cell leukemia virus type 1 infection of monocytes via STING-mediated apoptosis. Cell Host Microbe.

[17] Petrasek J., Iracheta-Vellve A., Csak T., Satishchandran A., Kodys K., Kurt-Jones E.A., Fitzgerald K.A., Szabo G. (2013). STING-IRF3 pathway links endoplasmic reticulum stress with hepatocyte apoptosis in early alcoholic liver disease. Proc. Natl. Acad. Sci. USA.

[18] Sanz-Garcia C., Poulsen K.L., Bellos D., Wang H., McMullen M.R., Li X., Chattopadhyay S., Sen G., Nagy L.E. (2019). The non-transcriptional activity of IRF3 modulates hepatic immune cell populations in acute on chronic ethanol administration in mice. J. Hepatol..

[19] Sanz-Garcia C., McMullen M.R., Chattopadhyay S., Roychowdhury S., Sen G., Nagy L.E. (2019). Nontranscriptional Activity of Interferon Regulatory Factor 3 Protects Mice From High-Fat Diet-Induced Liver Injury. Hepatol. Commun..

[20] Wang X., Majumdar T., Kessler P., Ozhegov E., Zhang Y., Chattopadhyay S., Barik S., Sen G.C. (2016). STING Requires the Adaptor TRIF to Trigger Innate Immune Responses to Microbial Infection. Cell Host Microbe.

[21] Lefrancois L., Lyles D.S. (1982). The interaction of antibody with the major surface glycoprotein of vesicular stomatitis virus. I. Analysis of neutralizing epitopes with monoclonal antibodies. Virology.

[22] Presloid J.B., Mohammad T.F., Lauring A.S., Novella I.S. (2016). Antigenic diversification is correlated with increased thermostability in a mammalian virus. Virology.

[23] Youseff B.H., Brewer T.G., McNally K.L., Izuogu A.O., Lubick K.J., Presloid J.B., Alqahtani S., Chattopadhyay S., Best S.M., Hu X. (2019). TRAF6 Plays a Proviral Role in Tick-Borne Flavivirus Infection through Interaction with the NS3 Protease. iScience.

[24] Mao H., Thakur C.S., Chattopadhyay S., Silverman R.H., Gudkov A., Banerjee A.K. (2008). Inhibition of human parainfluenza virus type 3 infection by novel small molecules. Antivir. Res..

[25] Martin C., Leyton L., Hott M., Arancibia Y., Spichiger C., McNiven M.A., Court F.A., Concha M.I., Burgos P.V., Otth C. (2017). Herpes Simplex Virus Type 1 Neuronal Infection Perturbs Golgi Apparatus Integrity through Activation of Src Tyrosine Kinase and Dyn-2 GTPase. Front. Cell. Infect. Microbiol..

[26] Subramanian G., Kuzmanovic T., Zhang Y., Peter C.B., Veleeparambil M., Chakravarti R., Sen G.C., Chattopadhyay S. (2018). A new mechanism of interferon’s antiviral action: Induction of autophagy, essential for paramyxovirus replication, is inhibited by the interferon stimulated gene, TDRD7. PLoS Pathog..

[27] Mizumoto N., Gao J., Matsushima H., Ogawa Y., Tanaka H., Takashima A. (2005). Discovery of novel immunostimulants by dendritic-cell-based functional screening. Blood.

[28] Kim T., Kim T.Y., Song Y.H., Min I.M., Yim J., Kim T.K. (1999). Activation of interferon regulatory factor 3 in response to DNA-damaging agents. J. Biol. Chem..

[29] Tewey K.M., Rowe T.C., Yang L., Halligan B.D., Liu L.F. (1984). Adriamycin-induced DNA damage mediated by mammalian DNA topoisomerase II. Science.

[30] Zhang S., Liu X., Bawa-Khalfe T., Lu L.S., Lyu Y.L., Liu L.F., Yeh E.T. (2012). Identification of the molecular basis of doxorubicin-induced cardiotoxicity. Nat. Med..

[31] Marques J.T., Rebouillat D., Ramana C.V., Murakami J., Hill J.E., Gudkov A., Silverman R.H., Stark G.R., Williams B.R. (2005). Down-regulation of p53 by double-stranded RNA modulates the antiviral response. J. Virol..

[32] Jiang R., Ye J., Zhu B., Song Y., Chen H., Cao S. (2014). Roles of TLR3 and RIG-I in mediating the inflammatory response in mouse microglia following Japanese encephalitis virus infection. J. Immunol. Res..

[33] Nacken W., Anhlan D., Hrincius E.R., Mostafa A., Wolff T., Sadewasser A., Pleschka S., Ehrhardt C., Ludwig S. (2014). Activation of c-jun N-terminal kinase upon influenza A virus (IAV) infection is independent of pathogen-related receptors but dependent on amino acid sequence variations of IAV NS1. J. Virol..

[34] Melchjorsen J., Rintahaka J., Soby S., Horan K.A., Poltajainen A., Ostergaard L., Paludan S.R., Matikainen S. (2010). Early innate recognition of herpes simplex virus in human primary macrophages is mediated via the MDA5/MAVS-dependent and MDA5/MAVS/RNA polymerase III-independent pathways. J. Virol..

[35] Fredericksen B.L., Keller B.C., Fornek J., Katze M.G., Gale M. (2008). Establishment and maintenance of the innate antiviral response to West Nile Virus involves both RIG-I and MDA5 signaling through IPS-1. J. Virol..

[36] Chazal M., Beauclair G., Gracias S., Najburg V., Simon-Loriere E., Tangy F., Komarova A.V., Jouvenet N. (2018). RIG-I Recognizes the 5′ Region of Dengue and Zika Virus Genomes. Cell Rep..

[37] Fensterl V., Sen G.C. (2009). Interferons and viral infections. Biofactors.

[38] Hiscott J. (2007). Triggering the innate antiviral response through IRF-3 activation. J. Biol. Chem..

[39] Zhu J., Smith K., Hsieh P.N., Mburu Y.K., Chattopadhyay S., Sen G.C., Sarkar S.N. (2010). High-throughput screening for TLR3-IFN regulatory factor 3 signaling pathway modulators identifies several antipsychotic drugs as TLR inhibitors. J. Immunol..

[40] Gall B., Pryke K., Abraham J., Mizuno N., Botto S., Sali T.M., Broeckel R., Haese N., Nilsen A., Placzek A. (2018). Emerging Alphaviruses Are Sensitive to Cellular States Induced by a Novel Small-Molecule Agonist of the STING Pathway. J. Virol..

[41] Gage Z.O., Vasou A., Gray D.W., Randall R.E., Adamson C.S. (2016). Identification of Novel Inhibitors of the Type I Interferon Induction Pathway Using Cell-Based High-Throughput Screening. J. Biomol. Screen..

[42] Probst P., Grigg J.B., Wang M., Munoz E., Loo Y.M., Ireton R.C., Gale M., Iadonato S.P., Bedard K.M. (2017). A small-molecule IRF3 agonist functions as an influenza vaccine adjuvant by modulating the antiviral immune response. Vaccine.

[43] Sali T.M., Pryke K.M., Abraham J., Liu A., Archer I., Broeckel R., Staverosky J.A., Smith J.L., Al-Shammari A., Amsler L. (2015). Characterization of a Novel Human-Specific STING Agonist that Elicits Antiviral Activity Against Emerging Alphaviruses. PLoS Pathog..

[44] Pang B., Qiao X., Janssen L., Velds A., Groothuis T., Kerkhoven R., Nieuwland M., Ovaa H., Rottenberg S., van Tellingen O. (2013). Drug-induced histone eviction from open chromatin contributes to the chemotherapeutic effects of doxorubicin. Nat. Commun..

[45] Taylor K.E., Mossman K.L. (2013). Recent advances in understanding viral evasion of type I interferon. Immunology.

[46] Luthra P., Aguirre S., Yen B.C., Pietzsch C.A., Sanchez-Aparicio M.T., Tigabu B., Morlock L.K., Garcia-Sastre A., Leung D.W., Williams N.S. (2017). Topoisomerase II Inhibitors Induce DNA Damage-Dependent Interferon Responses Circumventing Ebola Virus Immune Evasion. mBio.

[47] Pepin G., Nejad C., Ferrand J., Thomas B.J., Stunden H.J., Sanij E., Foo C.H., Stewart C.R., Cain J.E., Bardin P.G. (2017). Topoisomerase 1 Inhibition Promotes Cyclic GMP-AMP Synthase-Dependent Antiviral Responses. mBio.

[48] Zierhut C., Yamaguchi N., Paredes M., Luo J.D., Carroll T., Funabiki H. (2019). The Cytoplasmic DNA Sensor cGAS Promotes Mitotic Cell Death. Cell.

[49] Wang T., Yu N., Qian M., Feng J., Cao S., Yin J., Zhang Q. (2018). ERK-mediated autophagy promotes inactivated Sendai virus (HVJ-E)-induced apoptosis in HeLa cells in an Atg3-dependent manner. Cancer Cell Int..

[50] Wang F., Ma Y., Barrett J.W., Gao X., Loh J., Barton E., Virgin H.W., McFadden G. (2004). Disruption of Erk-dependent type I interferon induction breaks the myxoma virus species barrier. Nat. Immunol..

[51] Kaptein S.J., De Burghgraeve T., Froeyen M., Pastorino B., Alen M.M., Mondotte J.A., Herdewijn P., Jacobs M., de Lamballerie X., Schols D. (2010). A derivate of the antibiotic doxorubicin is a selective inhibitor of dengue and yellow fever virus replication in vitro. Antimicrob. Agents Chemother..

[52] Ash R.J., Diekema K.A. (1987). Inhibition of herpes simplex virus replication by anthracycline compounds. Antivir. Res..

[53] Shen L., Niu J., Wang C., Huang B., Wang W., Zhu N., Deng Y., Wang H., Ye F., Cen S. (2019). High-Throughput Screening and Identification of Potent Broad-Spectrum Inhibitors of Coronaviruses. J. Virol..

