# Supplementary material for: High Throughput Screening of FDA-Approved Drug Library Reveals the Compounds that Promote IRF3-Mediated Pro-Apoptotic Pathway Inhibit Virus Replication
Source: Viruses. 2020 Apr 14;12(4):442. doi: 10.3390/v12040442 (PMC7232324; doi:10.3390/v12040442)
Supplement: Supplementary file 1 [file viruses-12-00442-s001.zip › Suppl Fig.pdf]

			polyI:C + drugs											
			B1	B2	B3	B4	B5	B6	B7	B8	B9	B10		
polyI:C	Row-1	15486.25	11436.25	10155.25	11077.25	13662.25	12292.25	12870.25	9388.25	16937.25	13319.25	10763.25	862.25	NT
	Row-2	13526.25	11951.25	11067.25	12413.25	17445.25	11086.25	12362.25	10075.25	9971.25	11467.25	11726.25	1012.25	
	Row-3	13354.25	12284.25	12145.25	10721.25	11640.25	11014.25	13267.25	9957.25	10953.25	12448.25	9047.25	1027.25	
	Row-4	12222.25	11223.25	10586.25	10911.25	11959.25	22731.25	13016.25	12568.25	11746.25	11543.25	6871.25	852.25	
polyI:C / DMSO	Row-5	14813.25	12151.25	15527.25	8505.25	10944.25	10335.25	9665.25	12630.25	8629.25	9994.25	8630.25	0	Media
	Row-6	11695.25	13181.25	10188.25	7953.25	11021.25	9870.25	10748.25	11200.25	9588.25	10563.25	9631.25	0	
	Row-7	13836.25	11534.25	9406.25	10609.25	13729.25	11074.25	9637.25	12470.25	10987.25	10265.25	13302.25	0	
	Row-8	16112.25	9464.25	9926.25	9417.25	10110.25	11145.25	5084.25	10876.25	10080.25	11572.25	11490.25	0	
							Potential activator					Potential inhibitor		

Figure S1

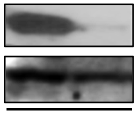

Figure S2

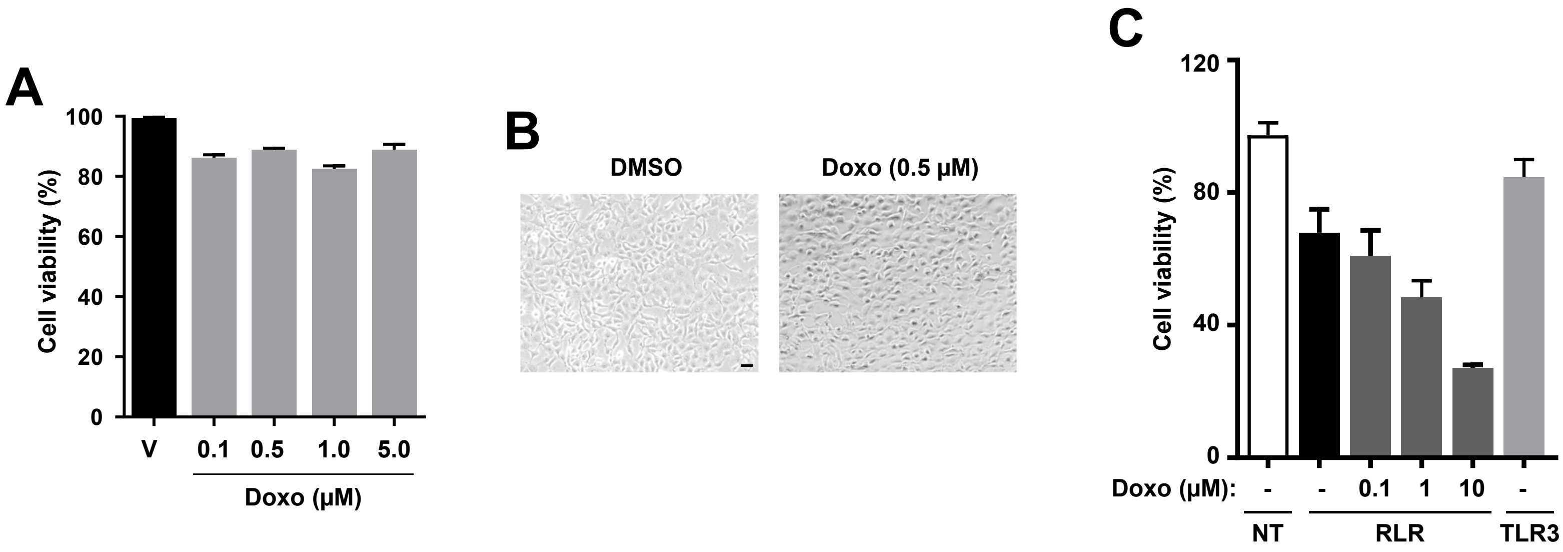
A
Cell viability (%)
0
20
40
60
80
100
V
0.1
0.5
1.0
5.0
Doxo (μM)
B
DMSO
Doxo (0.5 μM)
C
Cell viability (%)
0
40
80
120
Doxo (μM):
-
-
0.1
1
10
-
NT
RLR
TLR3

Figure S3
