# Supplementary material for: High Throughput Screening of FDA-Approved Drug Library Reveals the Compounds that Promote IRF3-Mediated Pro-Apoptotic Pathway Inhibit Virus Replication
Source: Viruses. 2020 Apr 14;12(4):442. doi: 10.3390/v12040442 (PMC7232324; doi:10.3390/v12040442)
Supplement: Supplementary file 1 [file viruses-12-00442-s001.zip › TABLE S1.pdf]

TABLE S1. Caspase activity of the primary screening plates. MDA-MB-453 cells were transfected with polyI:C in the absence or the presence of the drugs, as described in Fig 1F. Caspase-3 activity of each well is indicated after normalizing to the vehicle (DMSO) control, which was considered as 100.

Compounds	Caspase activity
Merbromin	3507.8184
Thioproperazine dimesylate	537.7076
Topotecan	470.294973
Perphenazine	408.521
Pyrvinium pamoate	378.4462014
Loperamide hydrochloride	351.1279
Nicergoline	321.3263
Oxandrolone	309.6642
Doxorubicin hydrochloride	305.4625
Acemetacin	292.5202
Proguanil hydrochloride	287.2808525
Canrenoic acid potassium salt	274.5111
Methotrexate	266.0738
Cyproheptadine hydrochloride	260.3053
Dantrolene sodium salt	258.95765
Pimethixene maleate	258.3525
Labetalol hydrochloride	256.72325
Mianserine hydrochloride	255.8156
Chenodiol	253.6044
N6-methyladenosine	253.30538
Vinpocetine	249.8804
Chloroxine	249.8664
Celecoxib	247.9434981
Disulfiram	247.9343
Isoconazole	246.5999
Clotrimazole	244.06165
Cyproterone acetate	242.3004988
Trimeprazine tartrate	241.1924
Gallamine triethiodide	239.08
Tetraethylenepentamine pentahydrochloride	236.276151
Cilnidipine	236.1445783
Alprenolol hydrochloride	235.49645
Cloperastine hydrochloride	234.9486
Glafenine hydrochloride	230.00355
Pergolide mesylate	226.83815
Benzydamine hydrochloride	224.7434
Desloratadine	223.6809306

Adenosine 5'-monophosphate monohydrate	223.64926
Econazole nitrate	222.2297
Pranoprofen	221.834914
Retinoic acid	221.7642
Chlormadinone acetate	221.4162943
Bumetanide	221.2987
Vardenafil	220.6896552
Zotepine	220.57
Trimipramine maleate salt	220.2116
Rifapentine	219.9575
Glibenclamide	219.9022
Methotrimeprazine maleat salt	218.3112
Loxapine succinate	217.5906
Triamcinolone	216.5064
Niflumic acid	216.13165
Fenoterol hydrobromide	216.0851
Gentamicine sulfate	214.4214
Zidovudine, AZT	214.34882
Secnidazole	214.180228
5-fluorouracil	213.7100125
Pirlindole mesylate	212.7535235
Glutethimide, para-amino	212.59385
Vincamine	210.5922
Norethindrone	209.70775
Ambroxol hydrochloride	208.90894
Quinidine hydrochloride monohydrate	208.68365
Guanfacine hydrochloride	207.19801
Naltrexone hydrochloride dihydrate	207.0683
Praziquantel	206.16995
Tamoxifen citrate	206.1231
Ifenprodil tartrate	204.77345
Cortisone	201.70115
Sulmazole	201.63379
Pirenzepine dihydrochloride	201.1469
Trimethobenzamide hydrochloride	200.8810573
Irinotecan hydrochloride trihydrate	200.614675
Chlorothiazide	199.37365
Dexamethasone acetate	199.3399
Rifaximin	198.0889074
Cyclopenthiazide	197.0780337
Carbetapentane citrate	196.93243

Bupivacaine hydrochloride	196.30135
Ethisterone	196.20825
Lisinopril	195.74275
Pioglitazone	195.7398
Amodiaquin dihydrochloride dihydrate	195.46345
Diazoxide	195.1421
Oxybutynin chloride	194.81175
Doxepin hydrochloride	194.579
Verapamil hydrochloride	194.5722
N-Acetyl-DL-homocysteine Thiolactone	194.401848
Methenamine	194.2667221
Chlorpheniramine maleate	194.044
Demecarium bromide	193.6245025
Flurandrenolide	192.439108
Erythromycin	192.237
Cinnarizine	191.8791
Ciclopirox ethanolamine	191.83575
Indomethacin	191.1343
Promethazine hydrochloride	190.6536
Pizotifen malate	190.6153317
Fludrocortisone acetate	190.52915
Triclosan	190.4445368
Fluvoxamine maleate	189.5152974
Busulfan	188.8593
Chlorprothixene hydrochloride	188.59713
Amitryptiline hydrochloride	186.1585903
Clozapine	185.43849
Amethopterin (R,S)	185.04366
Zuclopenthixol dihydrochloride	184.8401513
Nalbuphine hydrochloride	184.1472
Disopyramide	184.10525
Clindamycin hydrochloride	184.0499
Prednisolone	183.82595
Antazoline hydrochloride	183.63975
Flavoxate hydrochloride	183.5932
GBR 12909 dihydrochloride	183.24499
Amidopyrine	183.2437
Chlormezanone	183.20112
Desipramine hydrochloride	183.20112
Suxibuzone	182.889623
Losartan	182.6339842

Triprolidine hydrochloride	182.3829
Nafronyl oxalate	182.23598
Nefazodone HCl	182.01663
Nomifensine maleate	181.8954
Isotretinoin	181.49845
Bicalutamide	181.3872
Terfenadine	180.8946
Trazodone hydrochloride	180.66055
Picotamide monohydrate	180.6027
Pinacidil	180.5209
Citalopram Hydrobromide	180.451296
Ketotifen fumarate	180.34957
Famciclovir	179.9750727
Estropipate	178.858457
Mifepristone	178.752
Phentermine hydrochloride	178.479435
Telenzepine dihydrochloride	178.33305
Meloxicam	178.15607
Ibutilide fumarate	178.126204
Telmisartan	177.9808891
Fenofibrate	177.821
Dicloxacillin sodium salt hydrate	177.8
Dihydrostreptomycin sulfate	177.5864
Astemizole	177.5447
Dihydroergotamine tartrate	177.3779
Pindolol	177.2528
Carvedilol	176.8176153
Bepridil hydrochloride	176.75223
Methacycline hydrochloride	176.4523891
Ascorbic acid	175.96257
Neomycin sulfate	175.6126
Trifluridine	175.5922693
Ketoconazole	175.21678
Proadifen hydrochloride	174.393
6-Furfurylaminopurine	174.389449
Nocodazole	174.2087
Olmesartan	174.1587038
Enalapril maleate	173.86425
Butylscopolammonium (n-) bromide	173.85347
Flunisolide	173.596804
Tramadol hydrochloride	173.55151

Penbutolol sulfate	172.8491272
Procainamide hydrochloride	172.36523
Acetylsalicylsalicylic acid	172.1098
Fluoxetine hydrochloride	171.5943
Fleroxacin	170.6688824
Mepivacaine hydrochloride	169.8379726
Candesartan	169.8379726
Bifonazole	169.6717906
Etanidazole	169.39956
Nalmefene hydrochloride	169.1732447
Pempidine tartrate	169.052306
Phenylpropanolamine hydrochloride	168.50467
Debrisoquin sulfate	168.32919
Vecuronium bromide	168.0099709
Diphenidol hydrochloride	167.99895
Nicorandil	167.2031
Dizocilpine maleate	166.9946
Zaprinast	166.57439
Olopatadine hydrochloride	166.3481512
Pravastatin	166.3481512
Hexamethonium dibromide dihydrate	166.3259912
Clobetasol propionate	166.2606
Carprofen	166.1819693
Tacrine hydrochloride	166.17956
Clarithromycin	165.8496053
Clopamide	165.43377
Nadide	164.9322
Acyclovir	164.3814
Chlorthalidone	164.38089
Floxuridine	164.2145067
Pyrimethamine	164.0440529
Saquinavir mesylate	163.8715711
Caffeine	163.8554217
Methapyrilene hydrochloride	163.6351
Antipyrine, 4-hydroxy	163.6123348
Cisapride	163.5725
Sildenafil	163.5230577
Cladribine	162.8583299
Mepenzolate bromide	162.658303
Sulbactam	162.5259659
Lincomycin hydrochloride	162.41295

Paclitaxel	162.0462
Amoxicillin	161.8803
Chlorpropamide	161.79256
Asenapine maleate	161.391875
Moclobemide	161.26612
Ritodrine hydrochloride	161.17838
Granisetron	161.0303282
Aripiprazole	161.0303282
Vorinostat	160.7543641
Diethylcarbamazine citrate	160.6906
Cefmetazole sodium salt	160.476642
Pemirolast potassium	160.16937
Valacyclovir hydrochloride	159.635
Lansoprazole	159.5074813
Mexiletine hydrochloride	159.4803
Deptropine citrate	159.264352
Nandrolone	159.1268477
Cyclobenzaprine hydrochloride	158.2
Atorvastatin	158.0390528
Benfotiamine	158.008119
Montelukast	157.8728708
Chlorambucil	157.7618454
Ibandronate sodium	157.238121
Picrotoxinin	157.049396
Bezafibrate	157.01073
Tolnaftate	156.9162996
Hexetidine	156.891
Dimenhydrinate	156.73385
Venlafaxine	156.71724
Guanabenz acetate	156.5418
Benfluorex hydrochloride	156.44042
Warfarin	156.302045
Cefotaxime sodium salt	156.1943
Practolol	156.04559
Gatifloxacin	156.0448691
Oxethazaine	156.0088106
Betamethasone	155.56302
Methiothepin maleate	155.56302
Probucol	154.68562
Fluocinolone acetonide	154.3830494
Albendazole	154.22015

Ambrisentan	154.2168675
Medrysone	153.4302
Vidarabine	153.0738
Flunarizine dihydrochloride	152.91675
Baclofen (R,S)	152.6081
Clenbuterol hydrochloride	152.40438
Bisoprolol fumarate	152.18503
Racepinephrine HCl	152.0565019
Dropropizine (R,S)	151.89265
Tracazolate hydrochloride	151.83912
Risperidone	151.83912
S(-)Eticlopride hydrochloride	151.7148
Acetylsalicylic acid	151.557956
Imiquimod	151.391774
Acefylline	151.108333
Remoxipride Hydrochloride	150.8765899
Homochlorcyclizine dihydrochloride	150.822
Etoposide	150.78119
Rufloxacin	150.7270461
Enilconazole	150.464077
Brinzolamide	150.38636
Pentamidine isethionate	150.381
Methylprednisolone, 6-alpha	150.1703
(S)-(-)-Cycloserine	150.0311721
Zoxazolamine	149.46509
Carbimazole	149.45
Valproic acid	149.3975904
Bosentan	149.3975904
Cefoperazone dihydrate	149.07026
Diclazuril	148.8990445
Methylergometrine maleate	148.06125
Parbendazole	147.9114713
Zonisamide	147.862263
Clavulanate potassium salt	147.7357707
Benzathine benzylpenicillin	147.7139911
Diperodon hydrochloride	147.70315
Guanadrel sulfate	147.42
Ivermectin	147.2844
Alfuzosin hydrochloride	147.22772
Metergoline	147.22772
Ondansetron Hydrochloride	147.1633416

Etilefrine hydrochloride	147.0264696
Tremorine dihydrochloride	146.9645
Lidoflazine	146.78902
Triflupromazine hydrochloride	146.5814978
Domperidone	146.56967
Thioguanosine	146.39419
Sulfisoxazole	146.30645
Progesterone	146.3
Anastrozole	146.2401329
Amcinonide	145.907769
Oxfendazol	145.5139223
Fluorometholone	145.454132
Irsogladine maleate	145.227662
Sulfamerazine	145.031388
Didanosine	144.9492
Dipyridamole	144.8797
Pramipexole	144.4138879
Famotidine	144.3654
4-aminosalicylic acid	144.1708229
Furosemide	144.15682
Omeprazole	144.03375
Danazol	143.9484
Gemcitabine	143.9135854
Ethacrynic acid	143.6533
Thiamine hydrochloride	143.468745
Buspirone hydrochloride	143.4150395
Aprepitant	143.1756
Terconazole	143.1084
Cephalothin sodium salt	143.015805
Acetylcysteine	142.7503116
Clorgyline hydrochloride	142.53363
Niclosamide	142.2819383
Clofibrate	142.2517657
Meropenem	142.1758105
Cefsulodin sodium salt	141.9552
Levopropoxyphene napsylate	141.9264339
Ceforanide	141.855
Tetrahydrozoline hydrochloride	141.815514
Isradipine	141.8013063
Zardaverine	141.663802
Kanamycin A sulfate	141.65623

Bambuterol hydrochloride	141.56849
Nialamide	141.4276808
Nadifloxacin	141.4276808
Cyclophosphamide	141.4208558
Rosiglitazone Hydrochloride	140.9223099
Norcyclobenzaprine	140.895
Liothyronine	140.8516
Promazine hydrochloride	140.78885
Acetopromazine maleate salt	140.5715025
Ethamsylate	140.1512547
Ioxaglic acid	139.9314214
Trioxsalen	139.6565
THIP Hydrochloride	139.4637332
Butoconazole nitrate	139.2475
Clofazimine	139.0679
Milrinone	138.8092269
Benzylpenicillin sodium	138.5598504
Gliquidone	138.3636989
Sarafloxacin	138.3104738
Nitrocaramiphen hydrochloride	138.2261946
Levalbuterol hydrochloride	138.0610973
Fluconazole	137.7648525
Nimodipine	137.4032
Mefenamic acid	137.2687225
Loratadine	137.08984
Rivastigmine	136.9339427
Sulfacetamide sodic hydrate	136.9162996
Moxonidine	136.8511516
Nomegestrol acetate	136.8511516
Droperidol	136.61118
Pronethalol hydrochloride	136.3011344
Melatonin	136.1325
(S)-propranolol hydrochloride	136.0660848
Ibudilast	135.9368509
Guaiacol	135.8886215
Mebeverine hydrochloride	135.5536
Estradiol Valerate	135.4383049
Azapropazone	135.3386043
Oxiconazole Nitrate	135.293178
Cefdinir	134.939759
Doxofylline	134.304259

Butalbital	134.015
Nilvadipine	133.858868
Flucloxacillin sodium	133.8260571
Sulfadoxine	133.8216958
Tripelennamine hydrochloride	133.7764853
Fentiazac	133.5775
Cromolyn disodium salt	133.5604
Novobiocin sodium salt	133.486
Enoxacin	133.4441213
Toltrazuril	133.3229426
Fosinopril	133.2779393
Melengestrol acetate	133.1117574
Pyrilamine maleate	133.0399
Ranolazine	132.8241895
Mebhydroline 1,5-naphtalenedisulfonate	132.7025
Amiodarone hydrochloride	132.3
Mesalamine	132.2007481
Lynestrenol	132.1195
Salmeterol	131.6608
Raclopride	131.635
Canrenone	131.6259883
(R)-Propranolol hydrochloride	131.3279302
Minocycline hydrochloride	131.31755
Betaxolol hydrochloride	131.30291
Nicardipine hydrochloride	131.25904
Corticosterone	130.97
Haloperidol	130.7851
Chlortetracycline hydrochloride	130.6183
Alfaxalone	130.5259539
Talampicillin hydrochloride	130.5259539
Clodronate	130.484465
Naftifine hydrochloride	130.4551122
Gestrinone	130.3884496
Etidronic acid, disodium salt	130.262
Diflunisal	130.1938326
Moxifloxacin	129.9543
Tranilast	129.9471225
Apramycin	129.8384325
Arbutin	129.8384325
Bromhexine hydrochloride	129.621936
Lamotrigine	129.4557541

Homosalate	129.3329177
Pilocarpine nitrate	129.15
Ronidazole	129.0835411
Etomidate	128.9588529
Anethole-trithione	128.7910262
Bufexamac	128.7573
Ribavirin	128.7383981
Clonixin Lysinate	128.7094763
Niacin	128.686
Tribenoside	128.6008938
Benztropine mesylate	128.4586622
Mevastatin	128.2039525
Oxibendazol	128.1901725
Letrozole	128.1883809
Ethinylestradiol	128.1262983
Ciprofibrate	127.9613466
Probenecid	127.9401
Naftopidil dihydrochloride	127.9133723
Propoxycaine hydrochloride	127.8366584
Podophyllotoxin	127.818
Dyclonine hydrochloride	127.59355
Roxatidine Acetate HCl	127.365
Alprostadil	127.3633551
Mitotane	127.2953885
Meprylcaine hydrochloride	127.213217
Tulobuterol	127.1292065
Spironolactone	126.7958
Succinylsulfathiazole	126.679769
Oxacillin sodium	126.506142
Acebutolol hydrochloride	126.4052863
Oxaprozin	126.340399
Prothionamide	126.1258164
Flumethasone	126.0684
Sotalol hydrochloride	125.8508078
Balsalazide Sodium	125.5824
Trapidil	125.5757992
Terbutaline hemisulfate	125.4682
Prednicarbate	125.467581
Tetracycline hydrochloride	125.3502
Nifurtimox	125.3007906
Piperidolate hydrochloride	125.2182045

Docetaxel	124.9688409
Estriol	124.9688279
Itopride	124.6875
Benzbromarone	124.54693
Iopanoic acid	124.3453865
Imidurea	124.3453865
Ramipril	124.2206983
Furaltadone hydrochloride	123.9948
Alverine citrate salt	123.861275
3-alpha-Hydroxy-5-beta-androstan-17-one	123.826247
Protriptyline hydrochloride	123.7724
Cyclopentolate hydrochloride	123.7219451
Sertraline	123.2382262
Procarbazine hydrochloride	122.7244389
Fursultiamine Hydrochloride	122.688209
Oxytetracycline dihydrate	122.6127
Hydroxytacrine maleate (R,S)	122.5525
Ketanserin tartrate hydrate	122.44117
Piperacetazine	122.3503741
Bethanechol chloride	122.3503741
Topiramate	122.3503741
Bupropion hydrochloride	122.19375
Denatonium benzoate	122.1381918
Dimaprit dihydrochloride	122.1381918
Piperacillin sodium salt	122.0346
D-cycloserine	121.8516209
Alfadolone acetate	121.313166
Sulfinpyrazone	121.3093
Guanethidine sulfate	121.16965
Beta-Escin	121.153901
Cortisol acetate	121.138
Amifostine	121.1175
Pivampicillin	120.9006531
Azaperone	120.8541147
Nilutamide	120.5756
Clebopride maleate	120.46702
Memantine Hydrochloride	120.350636
Zalcitabine	120.350636
Penicillamine	120.323511
Nitrofurantoin	120.254475
Streptomycin sulfate	120.15993

Tiabendazole	120.0723
Oxyphenbutazone	119.8566085
Spaglumic acid	119.8566085
Mesna	119.8171998
Tazobactam	119.50067
Pivmecillinam hydrochloride	119.3578554
Minoxidil	119.339207
Phenylbutazone	119.2506016
Fexofenadine HCl	119.1084788
Pirenperone	118.947
Nortriptyline hydrochloride	118.84215
Clofibric acid	118.7946
Diphemanil methylsulfate	118.7753304
Dorzolamide hydrochloride	118.6097257
Pipenzolate bromide	118.1628
Halofantrine hydrochloride	118.1505672
Fluocinonide	118.0818
Althiazide	117.774
Ethopropazine hydrochloride	117.7008
Pepstatin A	117.3375
Moricizine hydrochloride	117.1134663
Imatinib	116.9921064
Clocortolone pivalate	116.9887781
Finasteride	116.971755
Adrenosterone	116.8608
Tosufloxacin hydrochloride	116.655
Metaproterenol sulfate, orciprenaline sulfate	116.65455
Flunixin meglumine	116.559
Neostigmine bromide	116.5325
Morpholinoethylamino-3-benzocyclohepta-(5,6-c)-pyridazine dihydrochloride	116.41
Drofenine hydrochloride	116.3322
Norgestimate	116.225507
Rabeprazole Sodium salt	116.1756
Guaifenesin	116.1378
Dehydrocholic acid	115.9538
Chloropyramine hydrochloride	115.8904
Rebamipide	115.665778
Dextromethorphan hydrobromide monohydrate	115.55358
Metrizamide	115.5525
Norgestrel-(-)-D	115.425
Anthralin	115.3302867

Alcuronium chloride	115.262977
Buflomedil hydrochloride	115.1325
Streptozotocin	114.7284
Lofexidine	114.669
Sulfamethizole	114.6312
Tridihexethyl chloride	114.6197007
Hycanthone	114.28135
Epiandrosterone	114.205
Carbadox	114.0312225
(-)-Isoproterenol hydrochloride	113.9962594
Benzamil hydrochloride	113.967253
Ozagrel hydrochloride	113.887934
Hydrocortisone base	113.8375
(+,-)-Synephrine	113.6221945
Bendroflumethiazide	113.5296
Methantheline bromide	113.1234414
Methiazole	113.1234414
Clioquinol	112.6428
Rofecoxib	112.59975
Tiletamine hydrochloride	112.512891
Rifampicin	112.3161
Meclofenoxate hydrochloride	112.1364
Tocainide hydrochloride	111.9628738
Cefadroxil	111.9125
Sulfadimethoxine	111.8448
Vatalanib	111.6325
2-Chloropyrazine	111.627182
Cefotetan	111.5625
Methacholine chloride	111.456
Antipyrine	111.4449339
Metoprolol-(+,-) (+)-tartrate salt	111.375
Sulfaquinoxaline sodium salt	111.0672
Colchicine	110.90336
Nadolol	110.6948
Formoterol fumarate	110.6771915
Tiratricol, 3,3',5-triiodothyroacetic acid	110.243475
Tylosin	110.147459
Minaprine dihydrochloride	110.1409692
Cefuroxime sodium salt	110.026675
Quetiapine hemifumarate	109.83
Megestrol acetate	109.6532

Zimelidine dihydrochloride monohydrate	109.6432
Fipexide hydrochloride	109.62525
Isoetharine mesylate salt	109.593
Norethynodrel	109.5154185
Betahistine mesylate	109.461
Sparfloxacin	109.3477358
Dicumarol	109.3014
Zileuton	109.166089
Isopyrin hydrochloride	109.0584
Molindone hydrochloride	108.3877621
Isoquinoline, 6,7-dimethoxy-1-methyl-1,2,3,4-tetrahydro, hydrochloride	108.2892
Closantel	108.15
Phenethicillin potassium salt	108.135
Dicyclomine hydrochloride	108.0881057
Flecainide acetate	108.0702
Clinafloxacin	108.0412
Bacampicillin hydrochloride	107.9225
Amikacin hydrate	107.9202
Deferoxamine mesylate	107.7786
Cefepime hydrochloride	107.7618454
Hesperidin	107.70085
Liranaftate	107.6976
Piretanide	107.5124688
Deoxycorticosterone	107.3148
Gabexate mesilate	107.2877277
Pentetic acid	107.2408
Fusidic acid sodium salt	107.17441
Quinethazone	107.138404
Rimantadine Hydrochloride	106.8587325
Sertaconazole nitrate	106.7643392
Phensuximide	106.5149626
Modafinil	106.4875
Misoprostol	106.3902743
Sulfanilamide	106.3854
Azlocillin sodium salt	106.3176
Piribedil hydrochloride	105.9126848
Nalidixic acid sodium salt	105.859627
Isoxsuprine hydrochloride	105.7004405
Sulconazole nitrate	105.6604
Fluvastatin sodium salt	105.6604
Azithromycin	105.5255505

Ticarcillin sodium	105.5174564
Ethionamide	105.49455
Pancuronium bromide	105.3626676
Carisoprodol	105.315
Sulfaguanidine	105.2246696
Fludarabine	105.1931865
Tolmetin sodium salt dihydrate	104.9908
Biotin	104.755
Iocetamic acid	104.4204
Azacytidine-5	104.2716
Leflunomide	104.2146
Perindopril	104.021197
Mevalonic-D, L acid lactone	103.9068
Meclozine dihydrochloride	103.88
Isometheptene mucate	103.8501203
Folinic acid calcium salt	103.8258
Hexylcaine hydrochloride	103.7448
Simvastatin	103.6764
Nitrendipine	103.5112
Monensin sodium salt	103.437
Sumatriptan succinate	103.300516
Sulfamethoxypyridazine	102.9186
Ethambutol dihydrochloride	102.7577093
Metyrapone	102.6972
Gabazine bromide	102.655
Fenoprofen calcium salt dihydrate	102.465
Isosorbide mononitrate	102.4002494
Methyldopate hydrochloride	102.337573
Mephentermine hemisulfate	102.0276
Tetracaïne hydrochloride	101.9737225
Rolipram	101.898
Isocarboxazid	101.8818
Nizatidine	101.8536
Dehydroisoandosterone 3-acetate	101.824
Clonidine hydrochloride	101.80485
Dichlorphenamide	101.7172
Quinacrine dihydrochloride dihydrate	101.71175
Lamivudine	101.71
Rimexolone	101.5125473
Pentobarbital	101.3191725
Mephenytoin	101.2780549

Sulfamethazine sodium salt	101.25
Torsemide	101.2375387
Crotamiton	101.2172
Clidinium bromide	100.9608
Bromopride	100.907483
Indoprofen	100.8616
Azathioprine	100.8306
Cetirizine dihydrochloride	100.79805
Opipramol dihydrochloride	100.764052
Tizanidine HCl	100.6715125
Propylthiouracil	100.59345
Methylhydantoin-5-(D)	100.5888
Miglitol	100.57485
Tioconazole	100.5248
Halcinonide	100.492288
Acitretin	100.446994
Acarbose	100.2374425
Thyroxine (L)	100.065
Benzthiazide	100.0432
Dimethadione	99.9068
Hymecromone	99.9
Ethaverine hydrochloride	99.882
Meptazinol hydrochloride	99.8624957
Brompheniramine maleate	99.855
Diphenylpyraline hydrochloride	99.458075
Dipyrone	99.450526
Alclometasone dipropionate	99.387
Theophylline monohydrate	99.2496
Methazolamide	99.2372
Famprofazone	99.186311
Phenacetin	99.11475
Dopamine hydrochloride	99.04445368
Triamterene	98.91629956
(-)-Emtricitabine	98.9145625
Carbidopa	98.7767625
(R) -Naproxen sodium salt	98.739
Carbenoxolone disodium salt	98.704
Quinapril HCl	98.6864
Acipimox	98.5011625
Methyldopa (L,-)	98.44428
Mometasone furoate	98.3289125

Phthalylsulfathiazole	98.2328
Tinidazole	98.0424
Ziprasidone Hydrochloride	97.7846025
Sulfapyridine	97.6536
Dolasetron mesilate	97.5764
Cefaclor hydrate	97.5663
Nifuroxazide	97.5663
Abacavir Sulfate	97.5616
Diltiazem hydrochloride	97.5363
Gefitinib	97.502884
Oxymetazoline hydrochloride	97.477675
Meglumine	97.458
Dacarbazine	97.4401025
Cinoxacin	97.3782
Glipizide	97.2861
Metixene hydrochloride	97.2594
Clobutinol hydrochloride	97.23
Dibenzepine hydrochloride	97.02
Ornidazole	97.001975
Theobromine	96.9556
Ethotoin	96.92916
Amfepramone hydrochloride	96.9108
Lithocholic acid	96.9084
Atractyloside potassium salt	96.8274
Clomiphene citrate (Z,E)	96.38239
Butenafine Hydrochloride	96.2550225
Homatropine hydrobromide (R,S)	96.12334802
Propofol	96.0964
Pyridostigmine iodide	96.0069825
Levocabastine hydrochloride	95.87487109
Pralidoxime chloride	95.8744
Glycopyrrolate	95.823
Urapidil hydrochloride	95.6968
Atropine sulfate monohydrate	95.5040125
(-) -Levobunolol hydrochloride	95.356
Ioversol	95.2816
Butacaine	95.2692
Benoxinate hydrochloride	95.24229075
Felodipine	95.2175
Cytarabine	95.1457325
Formestane	95.0436409

Sulfameter	94.9968
Dioxybenzone	94.8236
Hydralazine hydrochloride	94.566675
Decamethonium bromide	94.49982812
Phentolamine hydrochloride	94.445975
Entacapone	94.4291725
Viloxazine hydrochloride	94.4012
Oxantel pamoate	94.3205
Oxprenolol hydrochloride	94.29551122
Cloxacillin sodium salt	94.197475
Bemegride	94.1724
Isoxicam	94.06167401
Estrone	93.9208
Haloprogin	93.66
Alendronate sodium	93.6230425
Piroxicam	93.586875
Chlorotrianisene	93.474
Ribostamycin sulfate salt	93.4254
Gabapentin	93.31
S-(+)-ibuprofen	93.2696
Tobramycin	93.24645
Viomycin sulfate	93.17331671
Procyclidine hydrochloride	93.1612
Eucatropine hydrochloride	93.0366
Fosfosal	92.6528
Pentoxifylline	92.415375
Hemicholinium bromide	92.21474
Levonordefrin	92.178
Benzocaine	92.112898
Acetaminophen	91.9584
Primidone	91.9524
Methimazole	91.7568
Epitiostanol	91.61223788
Articaine hydrochloride	91.47473359
Diloxanide furoate	91.4344
Pyrazinamide	91.43295
Mafenide hydrochloride	91.378775
Loracarbef	91.3217825
2-Aminobenzenesulfonamide	91.2864
Propantheline bromide	91.2144
Zomepirac sodium salt	91.1412

Adamantamine fumarate	91.0525
Aminohippuric acid	91.0348
Carbinoxamine maleate salt	90.9788
Prazosin hydrochloride	90.9756
Naphazoline hydrochloride	90.86343612
Iodixanol	90.8548
Estradiol-17 beta	90.755
Bretylium tosylate	90.28
Alizapride HCl	90.125
(-)-MK 801 hydrogen maleate	90.0876
Troglitazone	89.9300025
Iopamidol	89.8752
Amphotericin B	89.7225
Tibolone	89.674775
(+) -Levobunolol hydrochloride	89.652
Zafirlukast	89.4890425
Repaglinide	89.43266833
Dinoprost trometamol	89.3375
Nylidrin	89.3172
Isopropamide iodide	89.3106
Piromidic acid	89.28
Metaraminol bitartrate	89.234575
Benperidol	89.1963
Diosmin	88.8827225
Flucytosine	88.8148
Flurbiprofen	88.7112
Thioperamide maleate	88.6816
Cefoxitin sodium salt	88.6476
Clemizole hydrochloride	88.538775
Quipazine dimaleate salt	88.5313325
Deflazacort	88.4864
Diprophylline	88.45814978
Aminopurine, 6-benzyl	88.382575
Bacitracin	88.1932
Tranylcypromine hydrochloride	88.134075
Ganciclovir	87.9284
Iproniazide phosphate	87.771975
Iopromide	87.7672
Mecamylamine hydrochloride	87.56234414
Nafcillin sodium salt monohydrate	87.3952
Iodipamide	87.3792

Ethosuximide	87.274975
Ciprofloxacin hydrochloride monohydrate	87.2364
Ampyrone	87.218175
Nifedipine	87.15418502
Paromomycin sulfate	87.0108
Captopril	86.99559471
Tenatoprazole	86.9397425
Oxalamine citrate salt	86.8868
Moxalactam disodium salt	86.6264
Ranitidine hydrochloride	86.273875
Levamisole hydrochloride	86.231275
N-Acetyl-L-leucine	86.2248
Lacidipine	86.1129425
Cefazolin sodium salt	86.0706
Pheniramine maleate	86.05286344
Imipramine hydrochloride	86.05286344
Azacyclonol	85.8464
Furazolidone	85.7212
Tetramisole hydrochloride	85.71345
Trihexyphenidyl-D,L Hydrochloride	85.666052
Benzonatate	85.65638767
Hydrochlorothiazide	85.6123348
Tetrahydroxy-1,4-quinone monohydrate	85.5352
Fenipentol	85.3068125
Felbinac	85.174
Cilostazol	85.1070025
Nimesulide	84.910675
Tranexamic acid	84.853875
Selegiline hydrochloride	84.8439
(R)-(+)-Atenolol	84.6116
Tomoxetine hydrochloride	84.5489125
Dobutamine hydrochloride	84.36201
Lorglumide sodium salt	84.3156
Betazole hydrochloride	84.24669604
Nisoldipine	84.058115
Ampicillin trihydrate	83.9977
Altretamine	83.9308
Metolazone	83.8865
Dapsone	83.77092511
Ethoxyquin	83.7378
Oxcarbazepine	83.7025

Terazosin hydrochloride	83.6406
Ropinirole HCl	83.5980925
Carbarsone	83.5275
Doxycycline hydrochloride	83.42334857
Salbutamol	83.398375
Etifenin	83.39775
Butamben	83.2842
Flutamide	83.256375
Troleandomycin	83.24229075
Pipemidic acid	83.2204
Scopolamine hydrochloride	83.1048
Methocarbamol	82.645775
Cephalosporanic acid, 7-amino	82.5475
Beclomethasone dipropionate	82.5468
Hydroflumethiazide	82.42290749
Pranlukast	82.352041
Orphenadrine hydrochloride	82.162975
Prilocaine hydrochloride	81.957075
(+)-Isoproterenol (+)-bitartrate salt	81.9328
Norfloxacin	81.893175
Suloctidil	81.795
Fluticasone propionate	81.7119285
Metronidazole	81.6903
Proglumide	81.580775
Temozolomide	81.5793225
Amrinone	81.5508
Miconazole	81.46255507
Sulfaphenazole	81.43612335
Spectinomycin dihydrochloride	81.4184
Phenformin hydrochloride	81.417475
Suprofen	81.406
Methylatropine nitrate	81.252
Isoniazid	81.197375
Trichlormethiazide	81.034
Amyleine hydrochloride	80.9339207
Hydroxyzine dihydrochloride	80.7729
Stavudine	80.7192
Levetiracetam	80.7180725
Xylazine	80.6491725
Mizolastine	80.4975925
Butylparaben	80.2308

Lidocaine hydrochloride	79.93832599
Midodrine hydrochloride	79.685075
Mebendazole	79.521775
Idoxuridine	79.49779736
Amoxapine	79.2022
Fadrozole hydrochloride	79.131275
Stanozolol	79.0125
Sulfachloropyridazine	78.924795
Heptaminol hydrochloride	78.8722467
Phenazopyridine hydrochloride	78.7644
Dimethisoquin hydrochloride	78.7475325
Aceclofenac	78.712375
Chlorzoxazone	78.648475
Nifenazone	78.627175
Etofylline	78.577475
Amlodipine	78.575
Gemfibrozil	78.250875
Aztreonam	78.201175
Dipivefrin hydrochloride	78.1825525
Timolol maleate salt	78.1588
Tropicamide	78.009475
Pentolinium bitartrate	77.768075
Flumequine	77.704175
Adiphenine hydrochloride	77.56828194
Oxolinic acid	77.469875
Ketorolac tromethamine	77.4632
Nisoxetine hydrochloride	77.4336
Chlorcyclizine hydrochloride	77.362152
Methylhydantoin-5-(L)	77.3512
Roxithromycin	77.3016
Maprotiline hydrochloride	77.25507
Ofloxacin	77.221375
Niridazole	77.0525
Prednisone	76.96035242
(+,-)-Octopamine hydrochloride	76.8416
Amiprilose hydrochloride	76.7064
Mercaptopurine	76.6254125
Mirtazapine	76.59945
Metformin hydrochloride	76.52863436
Phenelzine sulfate	76.419075
Tiapride hydrochloride	76.312575

Olanzapine	76.2972
Pargyline hydrochloride	76.120875
Atovaquone	75.81825
Pimozide	75.6903
Thiorphan	75.5092325
Yohimbine hydrochloride	75.3576525
Xylometazoline hydrochloride	75.325675
Thalidomide	75.162375
Monobenzone	75.1396
Idazoxan hydrochloride	75.0804
Nabumetone	75.0656
Ketoprofen	74.999075
Toremifene	74.9916
Pregnenolone	74.9115
Pefloxacine	74.3925
Diacerein	74.37
Tolazoline hydrochloride	74.2907489
Oxybenzone	74.1036
Cefixime	74.0425
Sulfadiazine	73.73568282
Erlotinib	73.6225
Lymecycline	73.59917497
Tolfenamic acid	73.380275
Spiramycin	73.3698
Fomepizole	73.3044325
Hydroxychloroquine sulfate	73.1804125
Acamprosate calcium	73.0081625
Ipsapirone	72.9117025
Doxazosin mesylate	72.8872
Nefopam hydrochloride	72.883275
Benazepril HCl	72.5947625
Zaleplon	72.4500725
Idebenone	72.415
Hexestrol	72.410008
Pyrantel tartrate	72.336575
Dydrogesterone	72.296773
Pentylenetetrazole	72.102275
Aceclidine Hydrochloride	72.0228925
Doxylamine succinate	71.92951542
Chlorphensin carbamate	71.782775
Lomefloxacin hydrochloride	71.569775

Sulfamethoxazole	71.534275
Dexfenfluramine hydrochloride	71.5130325
Flubendazol	71.4248
Josamycin	71.3626
Equilin	71.3248
Etofenamate	71.2305425
Aminophylline	71.1388
Exemestane	71.0720725
Penciclovir	70.9962825
Pramoxine hydrochloride	70.968149
Metoclopramide monohydrochloride	70.959175
Carbachol	70.928
Mephenesin	70.795875
Catharanthine	70.696475
Scopolamin-N-oxide hydrobromide	70.476375
Morantel tartrate	70.36123348
Sulindac	70.36123348
Mefexamide hydrochloride	70.078775
Testosterone propionate	69.72
Emedastine	69.3840225
Phenoxybenzamine hydrochloride	69.19
Thiamphenicol	69.13656388
Rifabutin	68.98379052
Argatroban	68.7570325
Meclofenamic acid sodium salt monohydrate	68.623275
Linezolid	68.6
Galanthamine hydrobromide	68.4883225
Griseofulvin	68.424475
L(-)-vesamicol hydrochloride	68.3908
Tropisetron HCl	68.39
Indapamide	68.381875
Naloxone hydrochloride	68.2351
Demeclocycline hydrochloride	68.121
Fenbufen	68.076575
Trimethadione	67.90395
Bephenium hydroxynaphthoate	67.8876
Ebselen	67.3758
Etodolac	67.196175
Diclofenac sodium	66.9656325
Urosiol	66.9404
Hyoscyamine (L)	66.862475

Camylofine chlorhydrate	66.8485025
Eburnamonine (-)	66.8416125
Carbamazepine	66.81938326
Antimycin A	66.784375
Riluzole hydrochloride	66.656575
Papaverine hydrochloride	66.5729025
(-)-Eseroline fumarate salt	66.49002494
Procaine hydrochloride	66.37004405
Trichlorfon	66.21145374
Clomipramine hydrochloride	65.4493
Spiperone	65.3562
Dibucaine	65.15418502
Reboxetine mesylate	64.9537525
Itraconazole	64.9261925
Propafenone hydrochloride	64.5885
Edrophonium chloride	64.5516
Ticlopidine hydrochloride	64.32599119
Tolbutamide	64.235475
Esmolol hydrochloride	63.7288
Fluspirilen	63.1368
Folic acid	63.0245525
Sertindole	62.5009125
Amprolium hydrochloride	62.20264317
Tolazamide	62.1426
Pridinol methanesulfonate salt	62.0622
Voriconazole	61.3847325
Fenbendazole	61.274775
Trimethoprim	61.267675
Levodopa	61.00440529
Darifenacin hydrobromide	60.94877965
Flufenamic acid	60.387275
Reserpine	60.2764
Carteolol hydrochloride	60.1125
Acetohexamide	59.77092511
Khellin	59.7561
Proscillaridin A	59.71124098
Fulvestrant	59.7005
Escitalopram	59.3315125
Allopurinol	58.9632
Ethynodiol diacetate	58.4304
Iohexol	58.1343

Meticrane	58.00881057
Todralazine hydrochloride	57.55066079
Pinaverium bromide	57.266714
Digoxin	56.98
Azelastine HCl	56.8442225
Hexachlorophene	56.66805152
Fenspiride hydrochloride	56.468075
Racecadotril	56.2516825
Nitrofural	55.22805
Vigabatrin	54.6282
Digitoxigenin	54.11
Proparacaine hydrochloride	53.6648
Pyridoxine hydrochloride	53.4819025
Dienestrol	53.3628
Acetazolamide	52.73127753
Chlorpromazine hydrochloride	52.55506608
Isosorbide dinitrate	52.450452
Sulpiride	52.35242291
Luteolin	51.8444
Amiloride hydrochloride dihydrate	51.55066079
Dequalinium dichloride	51.3279
Methoxamine hydrochloride	50.97795
Tyloxapol	50.69
Thimerosal	50.616045
Florfenicol	50.5272
Raloxifene hydrochloride	50.344
Chloramphenicol	49.9030837
Camptothecine (S,+)	48.785875
Naproxen	48.44052863
Biperiden hydrochloride	48.2019
Androsterone	48.16
Cimetidine	47.91189427
Amisulpride	47.6460725
Aminocaproic acid	47.508
Moxisylyte hydrochoride	47.34801762
Azaguanine-8	47.13656388
Calcipotriene	47.0225
Diflorasone Diacetate	46.7986025
Epirizole	46.55506608
Panthenol (D)	46.17621145
Thiostrepton	45.9234

Diphenhydramine hydrochloride	45.52422907
Serotonin hydrochloride	45.477
Bisacodyl	44.45
Allantoin	44.26431718
Moroxidine hydrochloride	44.2576
Eserine hemisulfate salt	43.9392525
Etretinate	43.9392525
Daunorubicin hydrochloride	43.37985
Benserazide hydrochloride	42.9348
Bromocryptine mesylate	42.6174
Efavirenz	41.965
Nicotinamide	41.4657425
Ifosfamide	41.3292
Sulfathiazole	41.18061674
Clemastine fumarate	40.26575
Alfacalcidol	39.0473525
Celiprolol HCl	38.7028525
Cefotiam hydrochloride	38.6508
Cyclosporin A	38.0975
Pyrithyldione	37.5224
(S)-(-)-Atenolol	37.4325
Dilazep dihydrochloride	36.098175
Isoflupredone acetate	34.51982379
Ethynylestradiol 3-methyl ether	34.0132
Thiethylperazine dimalate	33.69700748
Paroxetine Hydrochloride	33.6412
Dosulepin hydrochloride	32.94525
Molsidomine	32.90805
Zopiclone	32.3640525
Meclocycline sulfosalicylate	31.8325
Sulfamonomethoxine	31.6572
Bucladesine sodium salt	31.372
Etoricoxib	30.0559025
Digoxigenin	29.896
Bromperidol	29.70885
Aniracetam	29.6631725
Lanatoside C	29.471296
Tenoxicam	28.9881
Diethylstilbestrol	27.8964
Piracetam	27.7977
Dirithromycin	27.17925

Vancomycin hydrochloride	25.5285
Sulfasalazine	25.3053
Atracurium besylate	25.17180617
Imipenem	24.9054
Colistin sulfate	24.4683
Nystatine	23.16165
Mitoxantrone dihydrochloride	23.03175
Chlorhexidine	22.6153
Ceftazidime pentahydrate	22.4502
Iobenguane sulfate	22.4502
Budesonide	22.3758
Artemisinin	22.0503
Trimetazidine dihydrochloride	21.78525
Mefloquine hydrochloride	20.7666
Trolox	20.6925
Xamoterol hemifumarate	20.3796
Sisomicin sulfate	19.46955
Thiocolchicoside	19.251
Mesoridazine besylate	18.93945
Sibutramine HCl	18.786
Thioridazine hydrochloride	18.72246696
Ethamivan	18.5535
Clofilium tosylate	18.48035
Tiaprofenic acid	18.2931
Chloroquine diphosphate	17.3073
Fendiline hydrochloride	16.2925
Thonzonium bromide	15.5992
Cycloheximide	15.2604
Lovastatin	15.0009
Triflusal	14.77305
Phenindione	14.5731
Avermectin B1a	14.056238
Cyclizine hydrochloride	13.7826
Gliclazide	13.0014
Fluphenazine dihydrochloride	11.49785
Cyanocobalamin	11.41
Indatraline hydrochloride	10.89721554
Clorsulon	10.5555
DO 897/99	9.64875
Acenocoumarol	9.2721
Primaquine diphosphate	9.2575

Trifluoperazine dihydrochloride	8.70485
Prochlorperazine dimaleate	6.93146
Verteporfin	6.265586035
Propidium iodide	6.0912
Parthenolide	5.31495
R(-) Apomorphine hydrochloride hemihydrate	4.726
Prenylamine lactate	4.0176
Benzethonium chloride	2.184231887
Chicago sky blue 6B	2.17
Alexidine dihydrochloride	1.1664
Methyl benzethonium chloride	0.954713537
Auranofin	0.9052
Glimepiride	0
Sulfabenzamide	0
Perhexiline maleate	-0.3724
